# Unlocking the power of swine gut bacteria: newly isolated *Blautia* strain and its metabolites inhibit the replication of *Salmonella* Typhimurium in macrophages and alleviate DSS-induced colitis in mice

**DOI:** 10.1186/s40104-025-01208-7

**Published:** 2025-06-23

**Authors:** Jiatong Wei, Yang Liu, Hua Li, Ze Lu, Yanjiao Liu, Yifan Zhang, Cong Lan, Aimin Wu, Jun He, Jingyi Cai, Gang Tian, Daiwen Chen, Bing Yu, Zhiqing Huang, Ping Zheng, Xiangbing Mao, Jie Yu, Junqiu Luo, Hui Yan, Jiayong Tang, Huifen Wang, Quyuan Wang, Yuheng Luo

**Affiliations:** Key Laboratory for Animal Disease-Resistance Nutrition of Ministry of Education of China, Key Laboratory for Animal Disease-Resistance Nutrition and Feed of Ministry of Agriculture of China, Key Laboratory of Animal Disease-Resistant Nutrition of Sichuan Province, Engineering Research Center of Animal Disease-Resistance Nutrition Biotechnology of Ministry of Education of China, Animal Nutrition Institute, Sichuan Agricultural University, Chengdu, 611130 China

**Keywords:** *Blautia hominis*, Colitis, Inflammation, Macrophage

## Abstract

**Background:**

Inflammatory bowel disease is a significant health concern for both humans and large-scale farm animals. In the quest for effective alternatives to antibiotics, next-generation probiotics (NGPs) have emerged as a promising option. The genus *Blautia* presents a rich source of potential NGP strains. Here we successfully isolated *Blautia hominis* LYH1 strain from the intestines of healthy weaned piglets and characterized its biological traits. Its anti-inflammatory activity was then assessed using macrophages, while its protective effects against colitis and gut barrier damage were validated in a DSS-induced mouse colitis model.

**Results:**

*B. hominis* LYH1 displayed typical characteristics of an obligate anaerobe, including non-hemolytic and non-motile features, and a genome enriched with carbohydrate-active enzyme genes. It produced metabolites with antibiotic-like compounds, demonstrating antimicrobial activity against *Escherichia coli*. In vitro, *B. hominis* LYH1 effectively inhibited pathogen replication in macrophages, reducing cellular infections and alleviating inflammatory damage. In vivo, oral administration of *B. hominis* LYH1 or its metabolites significantly mitigated DSS-induced colitis in mice by suppressing pro-inflammatory cytokines, inhibiting T-lymphocyte activation, and enhancing short-chain fatty acid production.

**Conclusions:**

Our findings underscore *B. hominis* LYH1’s potential as a NGP for maintaining gut health and combating intestinal inflammation. These findings offer valuable insights into the development of antibiotic alternatives and innovative strategies for preventing and treating enteritis in both agricultural and medical settings.

**Supplementary Information:**

The online version contains supplementary material available at 10.1186/s40104-025-01208-7.

## Background

Inflammatory bowel diseases (IBD) constitute a substantial group of intestinal disorders that impact both humans and large-scale farm animals. These conditions are marked by a compromised intestinal barrier, abnormal immune activation, and microbial imbalance, or dysbiosis [1]. In the wake of restrictions on antibiotic use in animal feed, a variety of feed additives with potential antibiotic-replacement attributes have gained traction as pivotal research areas. Among these are probiotics, prebiotics, plant extracts, enzymes, organic acids, and antimicrobial peptides [2–4]. Probiotics, in particular, have emerged as a highly effective alternative to antibiotics. Defined as live microorganisms that confer health benefits to the host when consumed in sufficient quantities, probiotics include traditional, or first-generation probiotics (FGPs) [5], such as *Lactobacillus*, *Bacillus*, *Bifidobacterium* and yeast species, which primarily focus on sustaining host health. In contrast, next-generation probiotics (NGPs) comprise microorganisms isolated from animal intestines [6] and can be utilized therapeutically to manage host diseases, with many being strictly anaerobic. Their role extends past health maintenance to encompass disease control.

*Blautia*, a recently identified genus of intestinal bacteria, is instrumental in preserving the intestinal environment due to its antimicrobial and anti-inflammatory properties, effectively alleviating intestinal inflammation [7]. Numerous *Blautia* species boast robust biotransformation capabilities, producing flavonoid demethylases that transform polymethoxyflavonoids like apigenin, galanin, lignans, and quercetin into various demethylated metabolites [8]. Importantly, a decline in *Blautia* abundance has been correlated with obesity, metabolic syndrome, and IBD. Research indicates that IBD patients suffering from concurrent *Clostridium difficile* infections exhibit elevated levels of *Ruminococcus gnavus* and *Enterococcus*, coupled with diminished levels of *Blautia* [9]. Likewise, patients with Crohn’s disease (CD) [10] and colorectal cancer (CRC) [11] show significantly reduced levels of *Blautia* spp. in their intestinal mucosa compared to healthy individuals. Additionally, reduced *Blautia* abundance has been associated with intestinal inflammation in obese children [12]. Supplementation with *Blautia* has been shown to prevent intestinal inflammation by fostering Treg differentiation and the production of short-chain fatty acids (SCFAs) [13]. Moreover, specific *Blautia* strains have demonstrated protective effects against LPS-induced acute liver injury [14] and can improve disease symptoms by enhancing the pharmacological activity of drugs such as berberine for cholesterol reduction, thereby ameliorating dyslipidemia [15]. Consequently, *Blautia* is regarded as a valuable source of candidate strains for NGPs.

However, despite initial evidence of *Blautia*'s benefits, the precise strains suitable for NGPs application remain elusive. Current NGP research predominantly centers on humans and model animals, with a scarcity of clinically applicable products. Furthermore, most studies are confined to human isolates. Given the host-specific nature of probiotic functions, comprehensive investigations into the functions and mechanisms of animal isolates are crucial for developing strains tailored to animal intestinal health. Additionally, while research has explored the correlation between *Blautia* spp. abundance and IBD, these studies remain largely descriptive and lack profound mechanistic insights [7].

In light of the biological roles and limitations of *Blautia* spp., this study aimed to characterize the biological properties of *Blautia* strain isolated from the gut of healthy weaned piglets. An in vitro model of RAW264.7 cells was established with *Salmonella* Typhimurium as an intracellular infection agent to examine its regulatory effects on immune cells. Furthermore, an in vivo experiment was conducted to validate the protective effects of this strain against dextran sulfate sodium (DSS)-induced colitis in mice and to preliminarily elucidate the mechanisms by which it mitigates intestinal inflammatory damage. The ultimate objective of these findings is to provide new perspectives for the advancement of NGPs applications and the research and development of "alternative antagonism" strategies in animal production.

## Materials and methods

### Isolation and characterization of *B. hominis* LYH1 strain

Eighteen day-old healthy piglets from a commercial pig farm located in Ya'an, Sichuan (China) were selected, and fresh rectal content of each pig was aseptically collected. The collected samples were placed in an anaerobic bag and immediately transported to the laboratory for strain isolation. In an anaerobic chamber, 0.2 g of feces was mixed with 10 mL anaerobic PBS or enrichment medium (10^−^^1^ dilution), followed by serial tenfold dilutions to 10^−^^7^. From dilutions 10^−^^2^ to 10^−7^, 1 mL was inoculated into 9 mL enrichment medium in duplicate (*n* = 2) and incubated at 37 °C for 48–72 h. Single colonies with a diameter ≤ 1 mm, exhibiting milky-white pigmentation, a viscous consistency, and an opaque appearance were carefully picked. These colonies were then transferred into BHI medium tubes and incubated at 37 °C for 24–72 h. Bacterial DNA was extracted using the boiling method. The 16S rRNA gene was amplified with the universal primers 27F (5′-AGAGTTTGATCCTGGCCTCA-3′) and 1492R (5′-GGTTACCTTGTTACGACTT-3′). The PCR products were sent to Sangon Biotech (Shanghai) Co., Ltd. (Shanghai, China) for sequencing. The obtained sequences were compared with those in the NCBI database using the BLAST tool. After successful sequencing confirmation, the colonies were sub-cultured for strain preservation. The culture medium preparation referred to the described methods [16].

Purified bacterial cells were sent to Chengdu Lilai Biotechnology Co., Ltd. for scanning electron microscopy analysis of the bacterial surface’s three-dimensional structure. The Gram staining of isolated strain was carried out following the standard procedure [17]. Oxidase activity of the strain was tested with 1% p-phenylenediamine, and catalase activity was assessed by observing bubble formation with 3% hydrogen peroxide. Purified colonies were inoculated into anaerobic Brain-Heart Infusion (BHI, Hope Bio, Qingdao, China) broth, incubated at 37 °C, 180 r/min, for 12 h. Bubble formation indicated positive catalase activity. For gelatinase activity test, bacteria cultured for 18–24 h were stab-inoculated into gelatin deep tubes and incubated at 20–22 °C for 7–14 d, and liquefaction indicated a positive result. Bacteria were stained green with malachite green and counterstained red with safranin to check whether the strain produces spores. Bacterial suspension was stab-inoculated into semi-solid Gifu Anaerobic Medium (GAM, Hope Bio, Qingdao, China) and incubated anaerobically at 37 °C for 24 or 48 h. Non-diffusing growth indicated non-motility. Hemolytic activity of the strain was determined using blood agar plates with 5% sheep blood (Hope Bio, Qingdao, China). Bacterial suspension was spread onto the plates and incubated anaerobically at 37 °C for 48 or 72 h. Hemolysis was classified as β (clear halo), α (green halo), or γ (no halo). *Staphylococcus aureus* ATCC 25923 (American Type Culture Collection, VA, USA) was used as a positive control for β-hemolysis.

The purified strains were inoculated into modified GAM broth and incubated at 37 °C for 48 h. After incubation, DNA was extracted from the cultured strains employing the CTAB (Cetyltrimethylammonium bromide) method and stored at −20 °C for future use. The 16S rDNA gene fragment was amplified using the extracted DNA as a template with universal primers 27F (5′-AGAGTTTGATCCTGGCTCAG-3′) and 1492R (5′-GGCTACCTTGTTACGACTT-3′). The primers were synthesized, and the PCR products were sequenced by Sangon Biotech (Shanghai) Co., Ltd. The 16S rDNA sequences were analyzed using BLAST at the National Center for Biotechnology Information (NCBI), and sequences with a similarity greater than 98.65% were considered to be from the same species [18].

### Whole genome sequencing of *B. hominis* LYH1

Upon retrieving and reviving the frozen strain, it was inoculated into 100 mL of BHI liquid medium at a 1% inoculum volume and incubated for 24 to 48 h. The culture was subsequently centrifuged at 8,000 r/min for 5 min and dispatched to Shanghai Meiji Bio-pharmaceutical Technology Co., Ltd. for genome sequencing. Resistance gene composition analysis was performed in conjunction with the Comprehensive Antibiotic Resistance Database (CARD, http://arpcard.mcmaster.ca, version 1.1.3), considering only BLAST results with > 30% identity, ≥ 70% coverage, and an e-value cutoff of < 0.01. CAZymes were annotated using HMMER-3.1 (http://hmmer.org/) and compared against the CAZy database. Detailed CAZymes gene family classifications were obtained from the CAZy website (http://www.cazy.org/) based on these results. The raw data reported have been deposited in the Genome Sequence Archive (Genomics, Proteomics & Bioinformatics 2021) in National Genomics Data Center (Nucleic Acids Res 2022), China National Center for Bioinformation / Beijing Institute of Genomics, Chinese Academy of Sciences (PRJCA033624) that are publicly accessible at https://ngdc.cncb.ac.cn/gwh [19, 20].

### Drug susceptibility testing of *B. hominis* LYH1

Combining resistance genes distribution from whole genome sequencing, 16 commonly used antibiotics in animal production were selected to assess test strain resistance. These included β-lactams (ampicillin, amoxicillin, penicillin G, cefepime, and imipenem), aminoglycosides (streptomycin and gentamicin), tetracyclines (tetracycline), lincomycins (clindamycin and clarithromycin), polypeptides (vancomycin and polymyxin), quinolones (enrofloxacin), rifamycins (rifampin), sulfonamides (sulfadiazine), and oxazolidinones (linezolid), all of which were purchased from Sigma-Aldrich (Darm, Germany).

Drug susceptibility was evaluated using the paper disc diffusion method. Bacterial culture concentration was adjusted to 10^7^ CFU/mL in mid-logarithmic phase, spread onto BHI agar, and antibiotic discs were placed uniformly on the surface with three replicates per disc. Petri dishes were sealed in an anaerobic box with a gas-producing bag (MGC, Mitsubishi, Japan) and incubated at 37 °C for 48 to 72 h. Inhibition zone diameters were measured and compared to reference strains provided in the susceptibility disc instructions.

### Assessment of bacteriostatic activity via co-culture method

1. Metabolite extraction: After 48 h of culturing, the bacterial supernatant was harvested through centrifugation (8,000 r/min, 10 min). Metabolite extraction was performed in accordance with established protocols from previous research [21]. First, an equivalent volume of ethyl acetate was added to the supernatant, and the mixture was thoroughly agitated for 5 min. Subsequently, it was centrifuged at 8,000 r/min for 10 min to effectively separate the ethyl acetate layer containing the extracted metabolites. This layer was carefully transferred into a new tube, and the metabolites were concentrated using a nitrogen evaporator under a high-purity nitrogen atmosphere. After concentration, the dried metabolites were redissolved in 1 mL of culture medium. To guarantee purity, the solution was filtered through a 0.22-μm membrane (Sangon Biotech, Shanghai, China). Finally, the sample was stored at −20 °C for subsequent analysis.
2. Pathogen concentration adjustment: Three common pathogenic bacteria, *Escherichia coli* ATCC 25922 (American Type Culture Collection, VA, USA), *Staphylococcus aureus* ATCC 25923 (American Type Culture Collection, VA, USA), and *Salmonella* Typhimurium (preserved in our laboratory), were cultured to logarithmic growth phase in LB medium (Hope Bio, Qingdao, China). Subsequently, the bacterial cultures were centrifuged and resuspended in a saline solution. Their optical density (OD_600nm_) was adjusted to 0.5 for standardization purposes.
3. Bacteriostatic culture: The standardized bacterial suspensions were inoculated into LB medium containing the reconstituted metabolites. Each bacterial strain was set up with three replicate cultures. Each replicate comprised 10 mL of LB medium supplemented with 10 μL of the corresponding bacterial suspension. Subsequently, the cultures were incubated under agitated conditions (200 r/min) at 37 °C for 32 h. To track the bacterial growth kinetics, OD_600nm_ values were measured every 2 h, thereby generating comprehensive growth curves. Absorbance (OD_600nm_) was measured every 2 h to construct growth curves. Positive controls, consisting of LB medium inoculated with the respective pathogenic bacteria) and negative controls (LB medium alone) were incorporated. These controls served as benchmarks for evaluating the antibacterial efficacy of the metabolites.

To ensure consistency with the original metabolite concentration attained during the extraction process, the reconstituted metabolites were adjusted to the same volumes as that of the initial extraction solvent. The inhibition rate was calculated based on viable bacteria concentration changes post-oscillation between control and treated samples, using the following formula:

$$Y=\frac{W_t\;-\;Q_t}{W_t}\times100\%$$

Note: *Y* denotes the inhibition rate of metabolites. *W*_*t*_ represents the concentration of viable bacteria in the control sample (OD_600nm_). *Q*_*t*_ indicates the concentration of viable bacteria in the treatment sample (OD_600nm_). As per the China national standard GB/T 20944, an inhibition rate exceeding 70% indicates a bacteriostatic effect.

### Analysis of metabolite fractions in strain cultures

After activation, the test strain was inoculated into BHI medium at a 10% inoculum and incubated anaerobically at 37 °C for 72 h. The culture was centrifuged, and the supernatant was collected for analysis. Six samples, including three blank cultures and three supernatants from *B. hominis* LYH1 cultures, were sent to Shanghai Zhongke New Life Biotechnology Co., Ltd. for untargeted metabolomics analysis. The intricate procedures for sample processing and analysis adhered strictly to the methodologies outlined in our previous study [22]. The raw data reported have been deposited in the Genome Sequence Archive (Genomics, Proteomics & Bioinformatics 2021) in National Genomics Data Center (Nucleic Acids Res 2022), China National Center for Bioinformation/Beijing Institute of Genomics, Chinese Academy of Sciences (PRJCA033624) that are publicly accessible at https://ngdc.cncb.ac.cn/omix [19, 20].

### Preparation of live bacteria and metabolites, cell culture, and cell viability assay

After the activation of *B. hominis* LYH1, it was cultured until the logarithmic growth phase. The viable cell concentration was then adjusted to 10^6^ CFU/mL for subsequent use. Meanwhile, the activated strain was inoculated into sterile anaerobic BHI broth at an inoculum size of 10% (v/v) and then incubated at 37 °C for 72 h. After that, the culture was centrifuged at 8,000 r/min for 10 min. The resulting supernatant was collected and filtered through a 0.22-μm membrane for further applications.

The RAW264.7 cell line, obtained from the Cell Bank of the Institute of Cell Biology, Chinese Academy of Sciences (Shanghai), was cultured at 37 °C in a cell culture incubator with 5% carbon dioxide. These cells were grown in high-glucose DMEM medium supplemented with GlutaMAX™, 10% fetal bovine serum, and 1% penicillin-streptomycin, all of which were purchased from Gibco Life Technologies, Inc. The culture medium was replenished every 2–3 d to maintain optimal growth conditions until the cells reached 80%–90% confluence. For sub-culturing or harvesting, the cells were gently detached using 0.25% trypsin without EDTA (Gibco Life Technologies, MA, USA). Then, the cells were seeded in a 6-well plate at a density of 1 × 10^6^ cells/mL. After a 24-h incubation, the medium was replaced with an antibiotic-free medium for subsequent experimental procedures.

RAW264.7 cells were seeded in a 96-well plate, with each well receiving 100 μL of cell suspension. Once 80% confluency was achieved, various concentrations of live *B. hominis* LYH1 or its metabolites (diluted at 0.025 × , 0.05 × , and 0.1 ×) were added to the respective wells. Following a 24-h incubation, cell viability was evaluated using the CCK-8 assay kit (C0038, Beyotime Biotechnology, Shanghai, China). To perform this, the CCK-8 solution was dispensed added to each well and incubated for 2 h. The absorbance was then measured at 450 nm, providing a quantitative assessment of cell viability.

### Culture, processing and replication assay of *S.* Typhimurium

*S.* Typhimurium was inoculated into LB broth at a 1% inoculum and incubated at 37 °C with shaking until reaching mid-logarithmic growth. The concentration was adjusted to an MOI of 1:10 for cell infection and treated with varying concentrations (0.025 × , 0.05 × , 0.1 ×) of live bacteria or metabolites. After 1 h, extracellular bacteria were killed by replacing the medium with gentamicin-containing medium (50 µg/mL gentamicin). Cells were further incubated until mid-logarithmic growth. The phagocytosis assay used a *S.* Typhimurium strain expressing red fluorescent protein (RFP) under a constitutive promoter, which was preserved and provided by our laboratory.

Following previously described method [23], cell count and fluorescence intensity were measured using a CytoFlex flow cytometer (BD Biosciences, CA, USA), and data were analyzed with FlowJo 7.6 software.

### Animal trial, sample collection and blood index analysis

The animal trial was conducted in accordance with the rules for the care of laboratory animals (2017) formulated by the State Council of China, and approved by the Animal Care and Use Committee of Sichuan Agricultural University, China (SYXK-Chuan-2014-184).

Forty male C57BL/6J mice, at six-week-old, were randomly allocated into four experimental groups (*n* = 10 per group): CON (control), DSS (administered 3% DSS), B (treated with 3% DSS and *B. hominis* LYH1 at a concentration of 10^9^ CFU/mL), and B-M (exposed to 3% DSS alongside metabolites derived from *B. hominis* LYH1 at an equivalent concentration). This 28-day experiment was divided into two distinct phases. During the initial phase, spanning from d 1 to 21, the B and B-M groups received 0.2 mL of either *B. hominis* LYH1 or its corresponding metabolites via oral gavage every other day. In contrast, the CON and DSS groups were administered an equivalent volume of anaerobic sterile saline as a placebo. To obtain the metabolites, *B. hominis* LYH1 was first activated and then inoculated into anaerobic bottles containing BHI medium at an inoculum ratio of 10%. These cultures were incubated at 37 °C for 72 h, with optical density measurements at 600 nm (OD_600nm_) taken at regular intervals. After incubation, the cultures were centrifuged at 8,000 r/min for 15 min. The resulting supernatant was collected and aliquoted into 100-mL portions. These aliquots were then freeze-dried and re-constituted with sterile water to match the concentration of live bacteria (10^9^ CFU/mL), thus yielding a concentrated solution of bacterial metabolites. During the second phase (d 22–28), all gavage treatments were discontinued. All experimental groups, except the CON group, were provided with 3% DSS in their drinking water to induce acute colitis. The body weights of the mice were recorded every other day during the gavage period and daily during the DSS-induced attack period. The percentage change in body weight was calculated. Additionally, the disease activity index (DAI) was evaluated based on fecal consistency, rectal bleeding, and body weight changes. At the conclusion of the experiment, the mice were anesthetized and euthanized for sample collection. Blood was drawn from the heart and centrifuged (3,500 r/min, 30 min). The serum supernatant was aliquoted into 200-µL centrifuge tubes and stored at −20 °C for biochemical analysis. A 100-µL aliquot of whole blood was collected in anticoagulant tubes for routine hematological analysis and flow cytometry.

The abdominal cavity was opened, and the liver and spleen were carefully excised, weighed, and recorded, and the organ index was calculated using the following formula: Organ index (%) = [Organ fresh weight (g)/Fasting live weight before slaughter (g)] × 100%. The colon was removed by ligating both ends, and its length was measured and photographed. A proximal 1-cm segment of the colon was excised and fixed in 4% paraformaldehyde for histopathological analysis. The colonic contents were collected and stored at −80 °C for SCFAs analysis and 16S rRNA sequencing. The remaining colon tissue was stored at −80 °C for mRNA extraction and quantification, as well as the Western blot analysis.

After DSS treatment, the Disease Activity Index (DAI) was evaluated based on fecal scores and body weight changes, according to the scoring system (Table S1). The DAI was calculated as: DAI = (body mass index + stool shape + bleeding)/3.

To conduct the blood routine examination, 50 µL of fresh anticoagulated whole blood was analyzed using a blood cell analyzer (Myriad MC-80, Shenzhen, China) to determine white blood cell (WBC), red blood cell (RBC), neutrophil (Neu), monocyte (Mon), lymphocyte (Lym), eosinophil (Eos), platelet (PLT), and other blood cell counts.

Frozen serum samples were thawed at 4 °C, and the levels of complement C3 (C3), C-reactive protein (CRP), total protein (TP), immunoglobulin-G (IgG), immunoglobulin-M (IgM), triglycerides (TG), total cholesterol (TCH), lactate dehydrogenase (LDH), and albumin (ALB) were measured using an automated biochemistry analyzer (Hitachi 7020, Hitachi, Japan).

### Flow cytometry analysis of T lymphocyte subsets

According to the described method [24], 100 µL of fresh anticoagulated whole blood was used to label T lymphocytes (CD3^+^), helper T lymphocytes (CD3^+^CD4^+^), and cytotoxic T lymphocytes (CD3^+^CD8^+^). These antibodies were all purchased from BD Biosciences (CA, USA). The cells were analyzed using a CytoFlex flow cytometer (BD Biosciences, CA, USA), and the data were analyzed with FlowJo 7.6 software.

### Histopathological analysis of colonic tissue

The fixed colon segments underwent histopathological and immunohistochemical analysis by Wuhan Xavier Biotechnology Co., Ltd. Four random fields of view per section were examined microscopically, focusing on mononuclear and polymorphonuclear cell infiltration, epithelial proliferation and damage for scoring. Scores ranged from 0 to 3 [normal (0), mild (1), moderate (2) or severe (3)], with a total score ≥ 5 indicating typical colitis symptoms. Immunohistochemical analysis used Alcian Blue and Nuclear Solid Red staining, involving dehydration, then paraffin embedding, sectioning (8 μm), staining, and sealing. Mucus layer thickness was analyzed by selecting 10 fields of view. Images were analyzed using Image-pro plus 6.0 software (Media Cybernetics, MD, USA).

### Determination of SCFA concentrations in bacterial culture supernatant and colonic digesta

For supernatant preparation, test strains were activated and inoculated into BHI medium at a 10% inoculum ratio, incubated anaerobically at 37 °C for 72 h, and centrifuged at 8,000 × *g* for 10 min. One mL of supernatant was mixed with 0.2 mL of 25% metaphosphoric acid and 23.3 μL of 210 mmol/L crotonic acid, incubated at 4 °C for 30 min, and then centrifuged again at 8,000 × *g* for 10 min. The supernatant was filtered through a 0.22-μm membrane filter and stored. For colonic digesta preparation, 0.25 g of colonic content was homogenized with 0.6 mL of ultrapure water, left to stand at room temperature for 30 min, and then centrifuged at 1,000 × *g* for 15 min. Subsequently, 0.5 mL of the supernatant was mixed with 0.1 mL of 25% metaphosphoric acid and 11.65 μL of 210 mmol/L crotonic acid, and the remaining steps were the same as for bacterial supernatants. Acetic acid (AA), Propionic acid (PA), Butyric acid (BA), Valeric acid (VA), and Isovaleric acid (IsoVA) concentrations were determined using gas chromatography.

### Quantitative real-time PCR analysis

Total RNA was extracted from cells and colon samples using Trizol (Takara Biosciences, Beijing, China), following the instructions provided in the manual. PCR primers were designed using Premier 5.0, and details are presented in Table S2. Glyceraldehyde-3-phosphate dehydrogenase (*Gapdh*) and β-actin served as internal reference genes, with mRNA expression levels determined using the 2^−ΔΔCT^ method. The real-time PCR protocol and quantitative analysis were based on our published method [25].

### Western blot analysis

Approximately 0.1 g of colonic tissue was ground and lysed with RIPA Protein Lysate (Beyotime, Shanghai, China). The supernatant was centrifuged at 12,000 × *g* for 15 min at 4 °C and stored at −80 °C. Total protein concentration was measured using the BCA™ Protein Assay kit (Beyotime, Shanghai, China) according to the manufacturer’s instructions. Protein expression of TJP1 (Novus Biologicals, CO, USA), OCLN (Sigma-Aldrich, Darm, Germany) and CLDN1 (Sigma-Aldrich, Darm, Germany) in mouse colon was analyzed by Western blotting. In detail, treated samples (10 μg protein) were separated in a gel, and the membrane was transferred to a PVDF membrane at 100 V. The membrane was blocked with 5% BSA in TBST for 2 h, washed, and incubated with primary antibody overnight at 4 °C. After three TBST washes, the secondary antibody (Anti-mouse IgG, CST7076S, Cell Signaling Technology, Boston, MA, USA) was incubated for 1.5 h, followed by another three washes. Protein fluorescence was developed using the ECL Extreme Ultra-sensitive Luminescence Kit (Beyotime, Shanghai, China). Gray scale analysis was performed with Gel-Pro32 software, and target protein expression was calculated using GAPDH as an internal reference.

### 16S rRNA amplicon sequencing and bioinformatics analysis

Colonic digesta were collected and microbial diversity was assessed by 16S rRNA amplicon sequencing with 6 replicates. Genomic DNA was extracted using the E.Z.N.A Fecal DNA Kit (Omega Bio-Tek, GA, USA). High-throughput sequencing was performed by Novogene Co., Ltd. Extracted DNA was amplified using primers 341F (5'-TCCTACGGGGNGGCWGCAG-3') and 785R (5'-TGACTACHVGGGGTATCTAAKCC-3'). DNA libraries were constructed and sequenced on the Illumina MiSeq platform (CA, USA). Sequences were quality-controlled and denoised using DADA2 [26] in Qiime2 [27], removing chloroplasts and mitochondria sequences. Alpha and beta diversity analyses were performed, and differential microbial analyses used linear discriminant analysis of effect size (LEfSe; LDA > 3, *P* < 0.05). Correlations between SCFA concentrations and colonic microbiota abundance were analyzed with Spearman’s analysis. Data visualization was done using OmicShare Tools (https://www.omicshare.com/tools). The raw data reported have been deposited in the Genome Sequence Archive (Genomics, Proteomics & Bioinformatics 2021) in National Genomics Data Center (Nucleic Acids Res 2022), China National Center for Bioinformation / Beijing Institute of Genomics, Chinese Academy of Sciences (PRJCA033624) that are publicly accessible at https://ngdc.cncb.ac.cn/gsa [19, 20].

### Data processing and statistical analysis

Data analysis and visualization were conducted using Microsoft Excel 2019 and GraphPad Prism 9.4.1. SPSS 23 was used to assess data normality. For normally distributed data, statistical comparisons between two groups were performed using *t*-tests, and one-way ANOVA with Duncan's post hoc test was used for multiple group comparisons. Nonparametric tests were employed for non-normally distributed data to identify group differences. Results are expressed as mean ± standard error of the mean (SEM), with statistical significance set at *P* < 0.05. In figures and tables, statistical differences are denoted by *P < 0.05, **P < 0.01, and ***P < 0.001.

## Results

### Genome-wide analysis and identification of the porcine-derived *B. hominis* strain

**Fig. 1:**
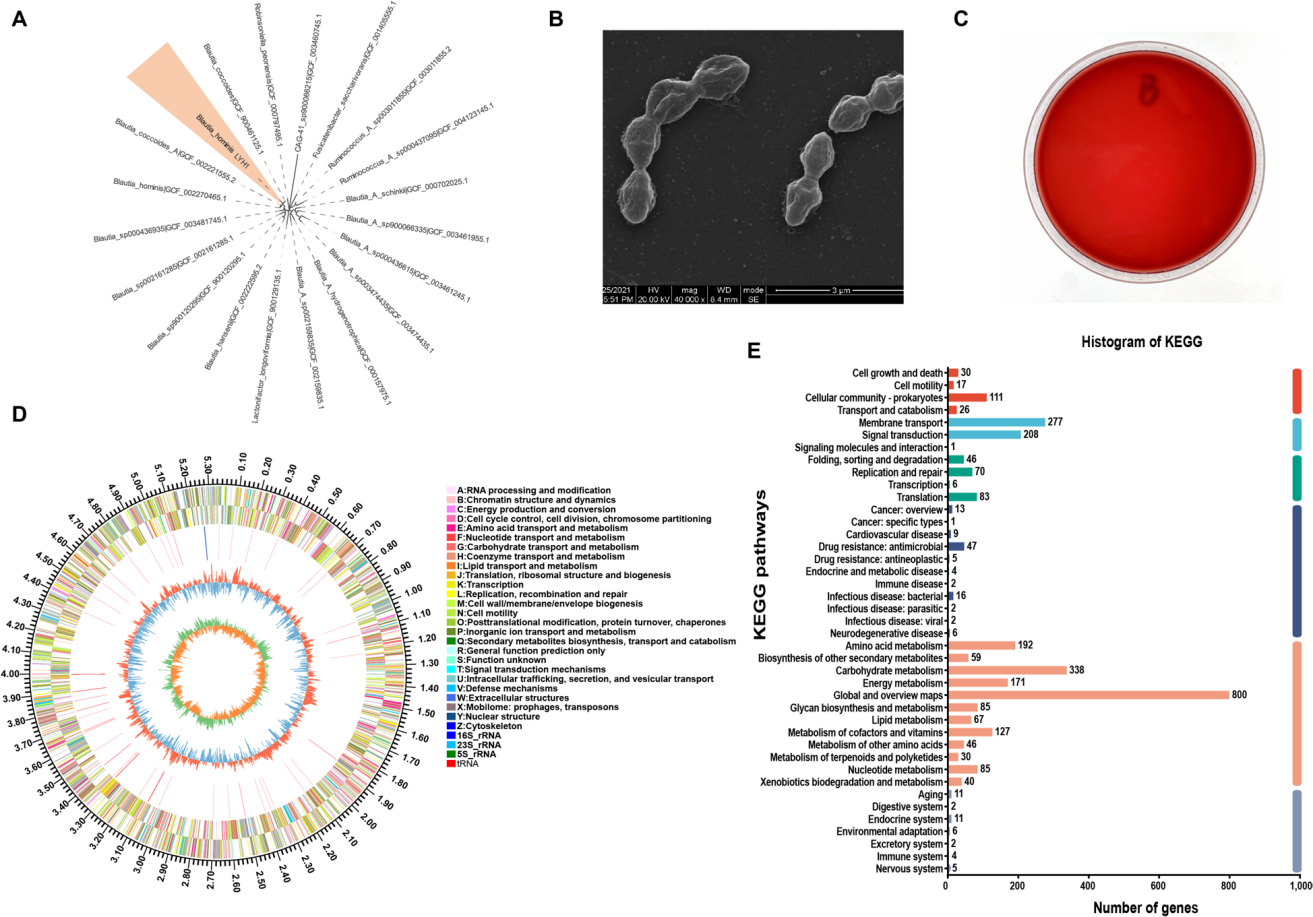
Morphological, hemolytic, and whole-genome of *B. hominis* LYH1. **A** Phylogenetic tree based on 16S rDNA. **B** Electron micrograph at 40,000 × magnification. **C** Growth pattern on blood agar plates. **D** Circular genome map. **E** KEGG clustering annotation (http://www.genome.jp)

The evolutionary tree analysis of 16S rDNA gene sequences (Fig. 1A) demonstrated that the 16S rRNA gene sequence similarity between the porcine-derived *B. hominis* strain and the *B. hominis* strain KB1, which is deposited in the NCBI GenBank database, reached 98.58%. Since this similarity failed to meet the 98.65% threshold, it was speculated that the isolated strain might represent a novel species within the *Blautia* genus, and thus it was temporarily designated as *B. hominis* LYH1. Phenotypically, *B. hominis* LYH1 exhibited characteristics such as Gram-positive staining, non-motility, negative results in catalase and oxidase tests, and absence of bacteriophage production (Table S3). The scanning electron microscopy of *B. hominis* LYH1 colonies (Fig. 1B) displayed an irregular cell surface with an oval-shaped morphology and no bacteriophage-like appearance. Additionally, the hemolysis testing of the bacterium yielded negative results (Fig. 1C).

To delve into the genetic and functional characteristics of *B. hominis* LYH1, a thorough genome analysis was carried out. The genome of *B. hominis* LYH1 comprised 6,572,367 base pairs, consisting of 121 contig assemblies with a GC content of 46.71%. It encoded 6,015 protein-coding genes, 56 small RNAs (sRNAs), one ribosomal RNA (rRNA), and 71 transfer RNAs (tRNAs). Moreover, it contained 230 tandem repeat sequences, totaling 70,045 base pairs (accounting for 1.19% of the genome), and 4,571 dispersed repeat sequences (0.08%), as shown in the genome map (Fig. 1D). Annotation against multiple databases, such as NR, Swiss-Prot, Pfam, COG, GO, and KEGG, identified 5,929, 3,962, 4,815, 4,521, 3,372, and 2,982 genes, respectively. KEGG pathway analysis indicated that 2,982 protein-coding genes were involved in 30 metabolic pathways, with the most significant ones being global and overview maps, carbohydrate metabolism, and amino acid metabolism (Fig. 1E). GO analysis highlighted transmembrane transport, DNA-templated transcription regulation, phosphorylation cascade signaling, and carbohydrate metabolic processes as the most abundant biological processes. The cellular components analysis revealed that the highest percentages of genes were related to membrane integral components, cytoplasmic membranes, and the cytoplasm. Molecular functions were predominantly enriched in DNA-binding and ATP-binding genes (Fig. S1A). COG database annotation classified these genes into 23 functional categories, with carbohydrate transport and metabolism, transcription, and signal transduction mechanisms emerging as key functions (Fig. S1B). These findings emphasize the crucial roles of these genes in material transport, energy conversion, information transfer, and carbohydrate metabolism.

The *B. hominis* LYH1 genome harbored 289 drug resistance genes, which were primarily distributed across various antibiotic classes, including macrolides, tetracyclines, fluoroquinolones, glycopeptides, and peptides (Table S4). In addition, the *B. hominis* LYH1 genome encoded 225 carbohydrate-active enzyme (CAZyme) genes, comprising glycoside hydrolases (GHs, 70.7%), glycosyl transferases (GTs, 11.6%), carbohydrate esterases (CEs, 13.8%), auxiliary activities (AAs, 3.6%), and polysaccharide lyases (PLs, 0.04%) (Fig. S1C). The GH family was the most gene-rich, with GH2 and GH109 being particularly abundant. These crucial enzymes play a significant role in the catabolism of CAZymes, such as β-galactosidase, β-mannosidase, β-glucuronidase, α-L-arabinosidase, and mannoprotein-β-mannosidase, underscoring their importance in carbohydrate metabolism.

### Analysis of major metabolites and biological characteristics of *B. hominis* LYH1 strain

**Fig. 2:**
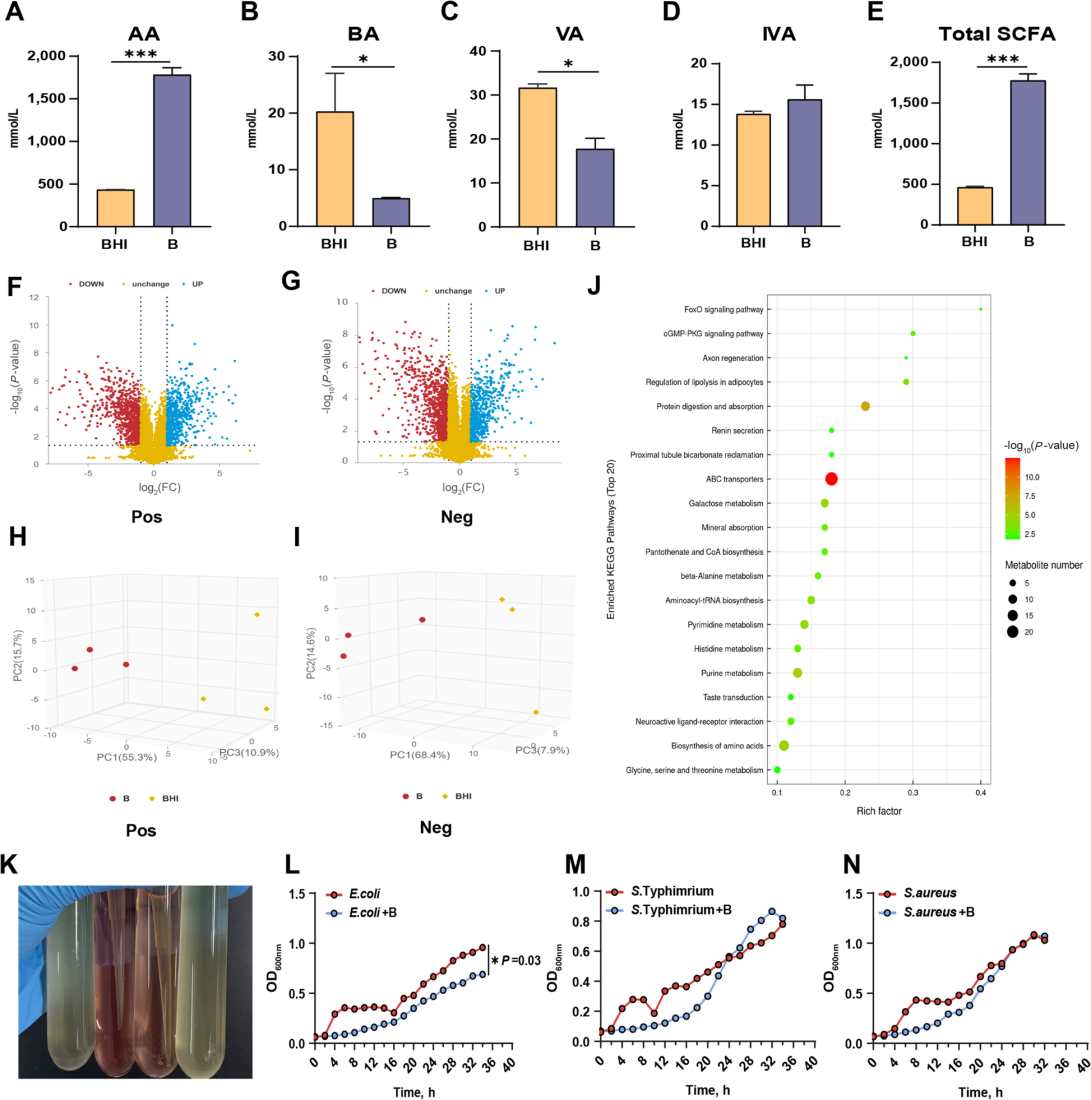
Metabolite profile of *B. hominis* LYH1 and antimicrobial activity of metabolites in co-cultures with pathogens. **A**–**E** Production of SCFAs (Acetic acid, Butyric acid, Valeric acid, Isovaleric acid, Total SCFA) in BHI medium. **F** and **G** Volcano plots of metabolite fold changes (|log_2_FC| > 1) and significance (*P* > 0.05) between B and BHI media (*n* = 3). **H** and **I** PCA plots of cationic and anionic metabolite profiles. **J** KEGG pathway enrichment analysis (*n* = 3). **K** The preparation and co-culture process of *B. hominis* LYH1 metabolites. The tubes on the left and right serve as positive controls with pathogens, while the middle two tubes contain the metabolite treatment group. **L**–**N** Growth curves of *E. coli*, *S.* Typhimurium, and *S. aureus* with and without the addition of metabolites (*n* = 3). BHI Blank BHI medium, B *B. hominis* LYH1. Error bars represent standard deviation, and asterisks denote statistical significance: ^*^P < 0.05, ^**^P < 0.01, and ^***^P < 0.001

As illustrated in Fig. 2A–E, acetic acid was the predominant SCFA produced by *B. hominis* LYH1 in BHI medium (*P* < 0.001), accompanied by a minor production of isovaleric acid (*P* = 0.381). After 72 h of fermentation, the SCFA production of *B. hominis* LYH1 increased approximately three-fold. To gain further insights into the metabolites of *B. hominis* LYH1, we employed a non-targeted metabolomics approach to determine the metabolite composition of the supernatant in BHI medium inoculated with and without *B. hominis* LYH1.

A comparison of *B. hominis* LYH1 culture supernatant with blank BHI media revealed an increase in metabolite identification in positive mode, with 352 metabolites upregulated and 920 downregulated (Fig. 2F). In negative mode, the respective numbers were 929 (upregulated) and 1716 (downregulated) (Fig. 2G). Principal component analysis (PCA) (Fig. 2H and I) further validated these metabolite differences between the control and *B. hominis* LYH1, which were also supported by KEGG metabolic pathway enrichment analysis (Fig. 2J). Notably, the top 20 KEGG pathways included ABC transporters, biosynthesis of amino acids, protein digestion and absorption, and purine metabolism. Utilizing both positive and negative ion modes, we screened for metabolites unique to *B. hominis* LYH1 based on stringent criteria: VIP value ≥ 1, multiplicity of difference ≥ 1, and *P* < 0.05. This approach yielded 102 exclusive metabolites, among which five exhibited antibiotic-like properties (Table S5). Notably, Methyl 3,4-O-isopropylmethylidene-D-lysine, 3,4-*O*-isopropylidene-D-lysine methyl ester (also mentioned redundantly as Methyl 3,4-*O*-isopropylidene-L-threonate, corrected for consistency), and 4-acetoxyphenol were the most abundant metabolites identified. These metabolites displayed varying sensitivities to antibiotics, with the highest abundance metabolite showing sensitivity to penicillin G, less sensitivity to vancomycin, and insensitivity to amoxicillin, streptomycin, clindamycin, cefepime, imipenem, clarithromycin, and gentamicin (Table S6).

Metabolites extracted using ethyl acetate were subsequently concentrated into a solid form using a nitrogen evaporator (Fig. 2K). In vitro co-culture experiments revealed that the culture supernatant of *B. hominis* LYH1 exerted a significant inhibitory effect on the growth of *E. coli* (Fig. 2L, *P* = 0.03). Nevertheless, no inhibitory effects were observed against *S.* Typhimurium (Fig. 2M, *P* = 0.46) or *S. aureus* (Fig. 2N, *P* = 0.41).

### Enhancement of macrophage viability and inhibition of *S. *Typhimurium infection by *B. hominis* LYH1 metabolites

**Fig. 3:**
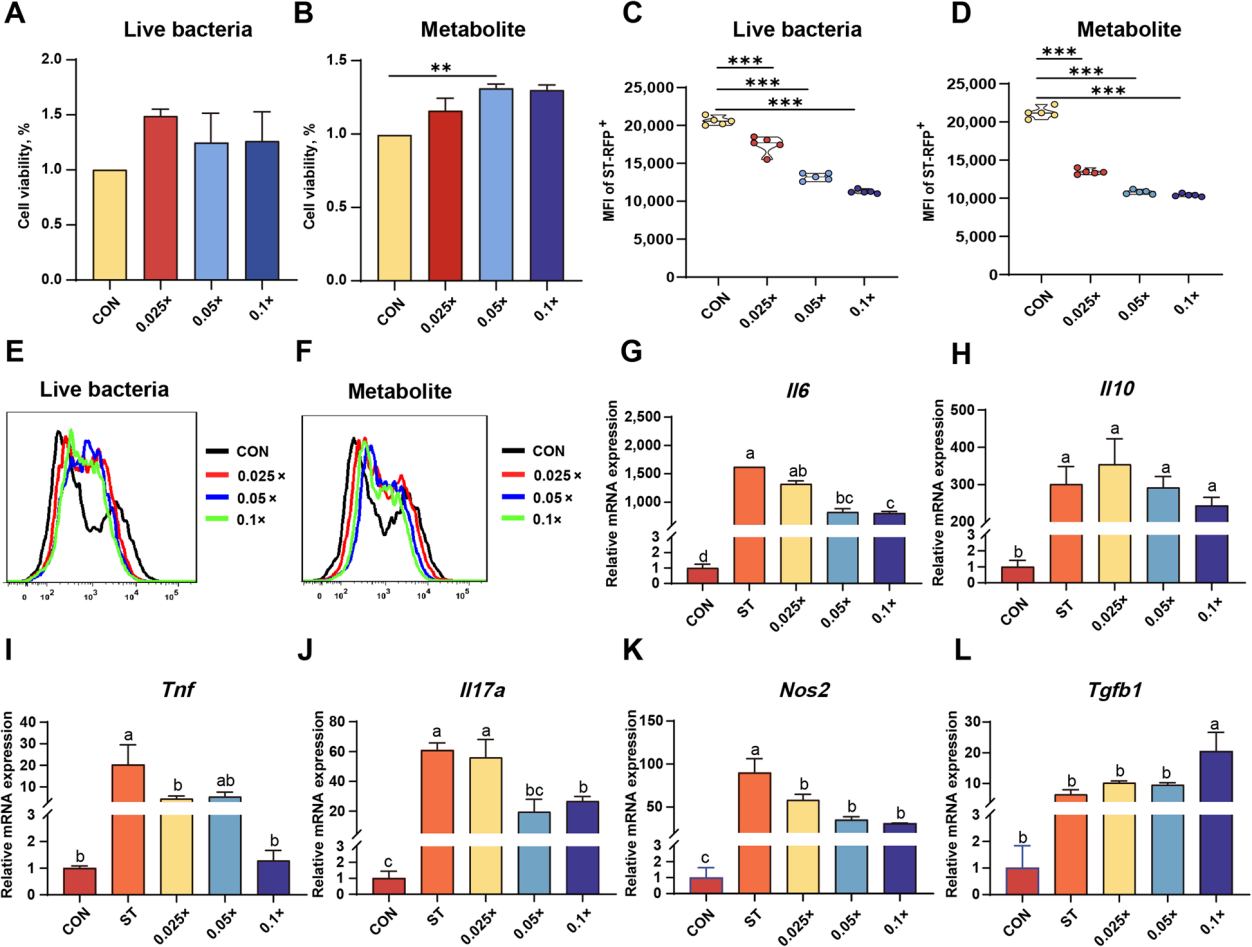
Effects of *B. hominis* LYH1 and metabolites on RAW264.7 macrophage viability, *S.* Typhimurium intracellular replication, and immune-related mRNA expression. **A** and **B** RAW264.7 macrophage viability with live bacteria or metabolites (*n* = 6). CON Control, 0.025×/0.05×/0.1× Respectively refer to live bacteria and their metabolites of* B. hominis* LYH1 at different concentrations. ^*^P < 0.05, ^**^P < 0.01. **C** and **D** Effects of live bacteria and metabolites at different concentrations on *S.* Typhimurium intracellular replication (*n* = 6). CON Control, 0.025×/0.05×/0.1× Respectively refer to live bacteria and their metabolites of *B. hominis* LYH1 at different concentrations. ^***^P < 0.001. **E** and **F** Flow cytometry histograms depicting the effects of *B. hominis* LYH1 live bacteria and metabolites on *S.* Typhimurium-infected cells, measured by ΔMFI (*n* = 6). CON Control, 0.025×/0.05×/0.1× Respectively refer to live bacteria and their metabolites of *B. hominis* LYH1 at different concentrations. **G–L** mRNA expression levels of *Il6*, *Il10*, *Tnf*, *Il17a*, *Nos2*, and *Tgfb1* in RAW264.7 macrophages treated with different concentrations of metabolites treatments (*n* = 6). CON Control, ST *S.* Typhimurium infection, 0.025×/0.05×/0.1× Cells infected with *S*. Typhimurium were treated with metabolites of *B. hominis* LYH1 at different concentrations. Identical lowercase letters indicate no significant difference between groups, while differing letters denote significant differences

None of the tested concentrations of live *B. hominis* LYH1 bacteria affected cell viability compared to the control group (Fig. 3A and B, *P* = 0.37). In contrast, the 0.05 × concentration of *B. hominis* LYH1 metabolites enhanced cell viability (*P* < 0.01), indicating that *B. hominis* LYH1 contamination results in no toxic effects on RAW264.7 cells. Further experiments involved infecting RAW264.7 macrophages with fluorescently labeled *S.* Typhimurium to assess the impact of live *B. hominis* LYH1 bacteria and its metabolites on the intracellular replication of *S.* Typhimurium. Flow cytometry results revealed that all concentrations of both live bacteria (Fig. 3C, *P* < 0.001) and metabolites (Fig. 3D, *P* < 0.001) reduced the total number of intracellular *S.* Typhimurium bacteria compared to the control group (*S.* Typhimurium-infected group). Additionally, the simultaneous administration of *B. hominis* LYH1 live bacteria and metabolites reversed the reduction in cell numbers observed in the *S.* Typhimurium-infected group (Fig. 3E and F). Notably, the inhibitory effect of *B. hominis* LYH1 metabolites on *S.* Typhimurium exhibited dose dependency, with 0.1 × concentration of *B. hominis* LYH1 metabolites demonstrating the highest reduction in intracellular *S.* Typhimurium replication by approximately 50% Mean Fluorescence Intensity (MFI) of ST-RFP^+^ (Fig. 3D).

The aforementioned results demonstrate that the metabolite group exerts a more potent inhibitory effect on *S.* Typhimurium infection of macrophages compared to the live bacteria group, as substantiated by further assays that measured the mRNA expression of inflammation-related cytokines and enzymes. Relative to the control group, *S.* Typhimurium infection induced the upregulation of mRNA expression of *Tnf*, *Nos2*, *Il6*, *Il10*, and *Il17a* in macrophages (Fig. 3G–K, *P* < 0.05). In contrast to the ST group, the 0.025 × concentration of LYH1 metabolites diminished the mRNA expression of *Tnf* and *Nos2* (Fig. 3I and K, *P* < 0.05), the 0.05 × concentration reduced the mRNA expression of *Il6*, *Il17a*, and *Nos2* (Fig. 3G, J, and K, *P* < 0.05), and the 0.1 × concentration downregulated the relative expression of *Il6*, *Tnf*, *Il17a*, and *Nos2* (Fig. 3G, I, J, and K, *P* < 0.05) while upregulating the expression of *Tgfb1* (Fig. 3L, *P* < 0.05).

### Influence of porcine-derived *B. hominis* LYH1 on intestinal damage and barrier function in mice with DSS-induced colitis

**Fig. 4:**
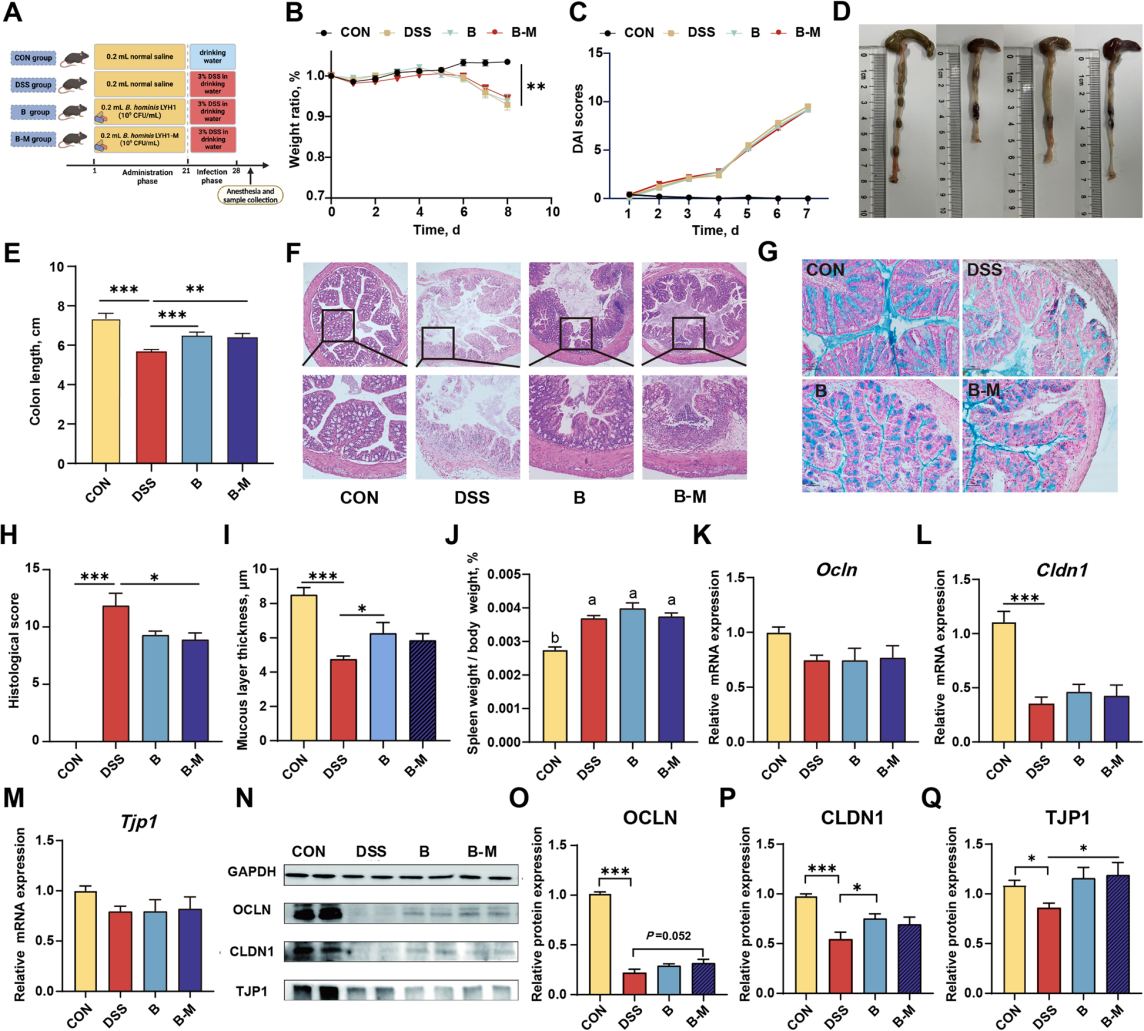
Effects of *B. hominis* LYH1 and metabolites on growth performance, colon length, spleen index, histopathological scores, and physical and chemical barriers in DSS-induced colitis mice. **A** Experimental design. **B** Body weight changes during DSS treatment (*n* = 10). **C** Disease Activity Index (DAI) scores (*n* = 10). **D** Representative images of cecum and colon from each group. **E** Colon length measurements across groups (*n* = 10). **F** Representative images of colonic morphology from each group, with hematoxylin and eosin (HE) staining; the upper row shows images at 4 × magnification, and the lower row shows images at 10 × magnification. **G** Representative images of colonic mucus layer thickness across groups, with Alcian Blue (AB) and nuclear fast red staining at 20 × magnification. **H** Histopathological scores of colonic tissues across groups (*n* = 10). **I** Measurements of colonic mucus layer thickness across groups (*n* = 10). **J** Spleen index (spleen weight/body weight) across groups (*n* = 10). **K–M** Relative mRNA expression levels of tight junction proteins *Ocln*, *Cldn1* and *Tjp1* in colonic tissues across groups (*n* = 10). **N** Western blot (WB) bands of tight junction proteins in colonic tissues across groups (*n* = 2). **O–Q** Protein expression levels of tight junction proteins OCLN, CLDN1 and TJP1 in colonic tissues across groups (*n* = 3, from two separate WB experiments). CON Control, DSS Administered 3% DSS, B Treated with 3% DSS and *B. hominis* LYH1 at a concentration of 10^9 ^CFU/mL, B-M Exposed to 3% DSS alongside metabolites derived from *B. hominis* LYH1 at an equivalent concentration. ^*^P < 0.05, ^**^P < 0.01, and ^***^P < 0.001. Identical lowercase letters indicate no significant difference between groups, while differing letters denote significant differences

Motivated by the anti-inflammatory and immunomodulatory capabilities of *B. hominis* LYH1 demonstrated in vitro, as previously described, we advanced to the subsequent phase of in vivo investigations to assess the protective efficacy of *B. hominis* LYH1 against DSS-induced colitis. Using a DSS-induced colitis mouse model, we observed that mice treated with DSS exhibited reduced body weights compared to the control (CON) group, accompanied by elevated disease activity index (DAI) scores (Fig. 4B and C, *P* < 0.05). Furthermore, DSS-induced colitis mice demonstrated markedly shorter colon lengths compared to the CON group (Fig. 4D and E, *P* < 0.05). Intriguingly, administration of both live *B. hominis* LYH1 and its metabolites increased colon length (Fig. 4E, *P* < 0.05).

Histological evaluations using hematoxylin and eosin (HE) staining and Alcian blue (AB) staining revealed that the CON group exhibited an intact mucosal epithelium with no notable epithelial cell degeneration or inflammatory cell infiltration (Fig. 4F and G). In contrast, DSS-induced colitis resulted in colonic mucosal ulceration, loss of mucosal epithelial and glandular structures, connective tissue hyperplasia, depletion of goblet cells, enhanced proliferation of the mucosa and submucosa, and considerable inflammatory cell infiltration. The histopathological scores of the DSS group’s colons were greater than those of the CON group (Fig. 4H, *P* < 0.05). However, gavage with viable *B. hominis* LYH1 and its metabolites mitigated these pathological scores (Fig. 4H, *P* < 0.05). The mucus layer thickness in the colon of DSS-treated mice was lesser than in the CON group, with only the administration of live *B. hominis* LYH1 significantly improving the thickness of the colonic mucus layer (Fig. 4I, P < 0.05).

The spleen index was increased in all DSS-exposed groups compared to the CON group (Fig. 4J, *P* < 0.05). Although we did not find any differences in the mRNA levels of *Ocln*, *Cldn1*, and *Tjp1* between the DSS and CON groups (Fig. 4K–M, *P* > 0.05), the abundance of proteins OCLN, CLDN1, and TJP1 in the DSS group showed decreased compared to the CON group (Fig. 4N–Q, *P* < 0.05). Compared to the DSS group, the protein abundance of CLDN1 in the *B. hominis* LYH1 treated group was greater than that in the DSS group (Fig. 4P, *P* < 0.05). In addition, the abundance of TJP1 protein in both live bacteria and its metabolite treated groups was greater than that in the DSS group (Fig. 4Q, *P* < 0.05).

### Effects of porcine-derived *B. hominis* LYH1 and its metabolites on immunity-related markers in mice with DSS-induced colitis

As shown in Table 1, compared to the CON group, the DSS group exhibited lesser concentrations of Mon, Eos, RBC, hemoglobin (HGB), and hematocrit (HCT) in blood (*P* < 0.05). Administration of live *B. hominis* LYH1 (B) and its metabolites (B-M) significantly increased Eos levels (*P* = 0.004). Mice in the DSS group had lesser concentrations of ALB, LDH, and TG in serum compared to the CON group (Table 2, *P* < 0.05), but exhibited elevated levels of C3, IgM, TBA, and TP (*P* < 0.01). Notably, mice in group B-M displayed reduced serum TBA levels compared to the DSS group (*P* < 0.001).

**Table 1:** Blood routine indices of mice in each group

Item	CON	DSS	B	B-M	*P*-value
WBC, 10^9^/L	4.04 ± 0.75	4.85 ± 1.68	4.64 ± 1.15	4.84 ± 2.80	0.507
Neu#, 10^9^/L	1.57 ± 0.49	1.81 ± 0.65	2.05 ± 0.67	2.15 ± 1.36	0.552
Lym#, 10^9^/L	2.16 ± 0.64	2.86 ± 1.09	2.33 ± 0.63	2.43 ± 1.47	0.608
Mon#, 10^9^/L	0.16 ± 0.09	0.10 ± 0.04	0.09 ± 0.04	0.13 ± 0.1	0.186
Eos#, 10^9^/L	0.15 ± 0.07	0.08 ± 0.04	0.17 ± 0.12	0.14 ± 0.11	0.093
Bas#, 10^9^/L	0.00 ± 0.00	0.01 ± 0.01	0.01 ± 0.01	0.00 ± 0.00	0.538
Neu%, %	38.84 ± 10.31	37.21 ± 6.34	43.8 ± 8.39	43.02 ± 1.00	0.249
Lym%, %	53.38 ± 12.57	58.91 ± 6.92	50.48 ± 10.85	50.69 ± 9.31	0.144
Mon%, %	4.00 ± 2.14^a^	1.99 ± 0.66^b^	1.94 ± 0.92^b^	2.78 ± 1.39^ab^	0.021
Eos%, %	3.75 ± 1.32^a^	1.71 ± 0.51^b^	3.69 ± 2.47^a^	3.43 ± 2.50^a^	0.004
Bas%, %	0.01 ± 0.03^b^	0.18 ± 0.23^a^	0.04 ± 0.05^ab^	0.08 ± 0.11^ab^	0.041
RBC, %	9.00 ± 0.48^a^	6.84 ± 0.56^b^	5.94 ± 1.28^c^	6.07 ± 1.17^bc^	<0.001
HGB, %	144.9 ± 7.02^a^	111.57 ± 8.79^b^	99.25 ± 19.00^b^	96.5 ± 25.75^b^	<0.001
HCT, 10^12^/L	40.76 ± 2.02^a^	31.62 ± 2.80^b^	28.03 ± 5.47^b^	28.63 ± 5.12^b^	< 0.001
MCV, g/L	45.32 ± 0.61^b^	46.17 ± 0.83^b^	47.38 ± 1.40^a^	47.29 ± 0.85^a^	< 0.001
MCH, %	16.1 ± 0.27^c^	16.29 ± 0.26^bc^	16.80 ± 0.47^a^	15.58 ± 0.40^ab^	0.001
MCHC, fL	355.2 ± 2.97	352.85 ± 8.94	354.38 ± 7.48	351.22 ± 6.40	0.459
RDW-CV, pg	12.41 ± 0.48^c^	13.54 ± 0.67^b^	14.78 ± 1.53^a^	14.20 ± 0.69^ab^	< 0.001
RDW-SD, g/L	24.93 ± 0.8^c^	27.82 ± 1.8^b^	31.01 ± 3.23^a^	30.00 ± 1.51^a^	< 0.001
PLT, %	187.1 ± 108.31^ab^	236.93 ± 97.22^a^	107.43 ± 22.63^b^	162.40 ± 45.36^ab^	0.006
MPV, fL	5.82 ± 0.41	5.95 ± 0.32	6.00 ± 0.36	5.86 ± 0.51	0.752
PDW, 10^9^/L	16.75 ± 0.45	16.73 ± 0.32	16.78 ± 0.24	16.72 ± 0.38	0.988
PCT, fL	0.11 ± 0.06^ab^	0.14 ± 0.06^a^	0.06 ± 0.02^b^	0.09 ± 0.02^ab^	0.007

*CON* Control, *DSS* Administered 3% DSS, *B* Treated with 3% DSS and *B. hominis* LYH1 at a concentration of 10^9^ CFU/mL, *B-M* Exposed to 3% DSS alongside metabolites derived from *B. hominis* LYH1 at an equivalent concentration, *WBC* White blood cell count, *Neu#* Neutrophil absolute count, *Lym#* Lymphocyte absolute count, *Mon#* Monocyte absolute count, *Eos#* Eosinophil absolute count, *Bas#* Basophil absolute count, *Neu%* Neutrophil percentage, *Lym%* Lymphocyte percentage, *Mon%* Monocyte percentage, *Eos%* Eosinophil percentage, *Bas%* Basophil percentage, *RBC* Red blood cell count, *HGB* Hemoglobin, *HCT* Hematocrit, *MCV* Mean corpuscular volume, *MCH* Mean corpuscular hemoglobin, *MCHC* Mean corpuscular hemoglobin concentration, *RDW-CV* Red cell distribution width-coefficient of variation, *RDW-SD* Red cell distribution width-standard deviation, *PLT* Platelet count, *MPV* Mean platelet volume, *PDW* Platelet distribution width, *PCT *Plateletcrit. Data are presented as mean ± SEM

^a,b,c^Means within the same row with different superscript letters are significantly different (*P* < 0.05)

**Table 2:** Serum biochemical indices of mice in each group

Item	CON	DSS	B	B-M	*P*-value
ALB, g/L	16.16 ± 5.56^a^	12.26 ± 1.01^b^	11.38 ± 1.08^b^	12.25 ± 2.61^b^	0.019
C3, g/L	0.13 ± 0.03^b^	0.21 ± 0.05^a^	0.19 ± 0.05^a^	0.18 ± 0.03^a^	0.003
CRP, mg/L	2.39 ± 0.65	2.32 ± 0.38	2.43 ± 0.34	2.37 ± 0.25	0.933
IgG, g/L	1.17 ± 0.38	1.23 ± 0.52	1.02 ± 0.28	1.21 ± 0.4	0.646
IgM, g/L	0.05 ± 0.06^b^	0.28 ± 0.08^a^	0.26 ± 0.07^a^	0.33 ± 0.08^a^	< 0.001
TBA, µmol/L	1.36 ± 0.54^c^	27.42 ± 8.38^a^	21.94 ± 2.41^ab^	18.98 ± 5.35^b^	< 0.001
LDH, U/L	680.08 ± 657.98^a^	257.47 ± 158.04^b^	153.46 ± 83.58^b^	174.36 ± 68.99^b^	0.034
TBiL, µmol/L	0.86 ± 0.91	1.40 ± 0.88	1.43 ± 0.56	1.13 ± 0.70	0.475
TC, mmol/L	2.47 ± 0.40	2.73 ± 0.43	2.61 ± 0.25	2.56 ± 0.30	0.542
TG, mmol/L	1.94 ± 0.44^a^	0.57 ± 0.16^b^	0.52 ± 0.09^b^	0.47 ± 0.06^b^	0.002
TP g/L	39.69 ± 7.34^b^	55.71 ± 3.98^a^	53.22 ± 2.72^a^	52.49 ± 4.42^a^	< 0.001

*CON* Control, *DSS* Administered 3% DSS, *B* Treated with 3% DSS and *B. hominis* LYH1 at a concentration of 10^9^ CFU/mL, *B-M* Exposed to 3% DSS alongside metabolites derived from *B. hominis* LYH1 at an equivalent concentration, *ALB* Albumin, *C3* Complement C3, *CRP* C-reactive protein, *IgG* Immunoglobulin G, *IgM* Immunoglobulin M, *TBA* Total bile acids, *LDH* Lactate dehydrogenase, *TBiL* Total bilirubin, *TC* Total cholesterol, *TG* Triglycerides, *TP* Total protein. Data are presented as mean ± SEM

^a,b,c^Means within the same row with different superscript letters are significantly different (*P* < 0.05)

**Fig. 5:**
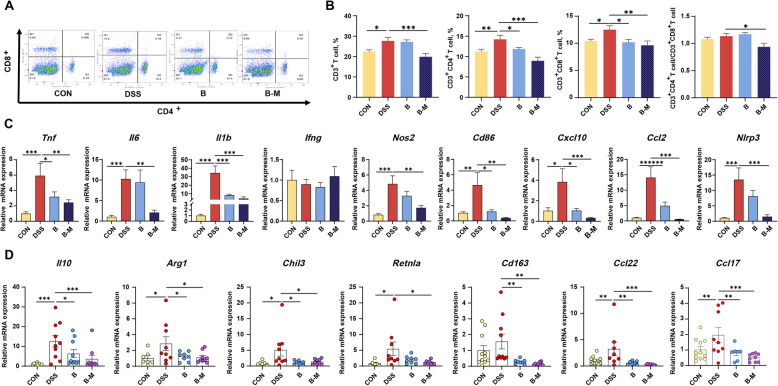
Effects of *B. hominis* LYH1 and metabolites on T lymphocytes and immune gene expression in DSS-colitis mice. **A** Representative flow cytometry plots from each group. **B** Proportions of T lymphocyte subsets, including CD3^+^, CD3^+^CD4^+^, CD3^+^CD8^+^, and the CD3^+^CD4^+^/CD3^+^CD8.^+^ ratio (*n* = 10). **C** and **D** Relative expression levels of pro-inflammatory and anti-inflammatory cytokine genes in the colons of mice across groups (*n* = 10). CON Control, DSS Administered 3% DSS, B Treated with 3% DSS and *B. hominis* LYH1 at a concentration of 10^9^ CFU/mL, B-M Exposed to 3% DSS alongside metabolites derived from *B. hominis* LYH1 at an equivalent concentration.^*^P < 0.05, ^**^P < 0.01, and ^***^P < 0.001

According to the flow cytometry analysis of T lymphocyte subpopulations in blood (Fig. 5A), the proportions of CD3^+^, CD3^+^CD4^+^ T cells, and CD3^+^CD8^+^ T cells were elevated in the DSS group compared to the CON group (Fig. 5B, *P* < 0.05), while mice in groups B and B-M demonstrated reduced proportions of CD3^+^CD4^+^ T cells and CD3^+^CD8^+^ T cells compared to the DSS group (Fig. 5B, *P* < 0.05). Additionally, the proportion of CD3^+^ T cells and the ratio of CD3^+^CD4^+^ T cells to CD3^+^CD8^+^ T cells were decreased in group B-M compared to the DSS group (Fig. 5B, *P* < 0.05).

Furthermore, compared to the CON group, the mRNA levels of pro-inflammatory factors, including *Tnf*, *Il6*, *Il1b*, *Nos2*, *Cd86*, *Cxcl10*, *Ccl2*, and *Nlrp3*, as well as anti-inflammatory factors such as *Il10*, *Arg1*, *Chil3*, *Retnla*, *Cd163*, *Ccl17*, and *Ccl22* in the colonic tissues of mice in DSS group were upregulated (Fig. 5C and D, *P* < 0.05). Compared to the DSS group, the mRNA levels of *Tnf*, *Il1b*, *Cd86*, *Cxcl10*, *Ccl2*, *Il10*, *Arg1*, *Chil3*, *Cd163*, *Ccl17*, and *Ccl22* showed decreased in the colonic tissues of mice in B and B-M groups (Fig. 5C and D, *P* < 0.05). Notably, the mRNA levels of *Il6*, *Nos2*, *Retnla*, and *Nlrp3* were specifically reduced of mice in group B (Fig. 5C and D, *P* < 0.05).

### Influence of porcine-derived* B. hominis* LYH1 and its metabolites on SCFA production and intestinal microbiota in mice with DSS-induced colitis

**Fig. 6:**
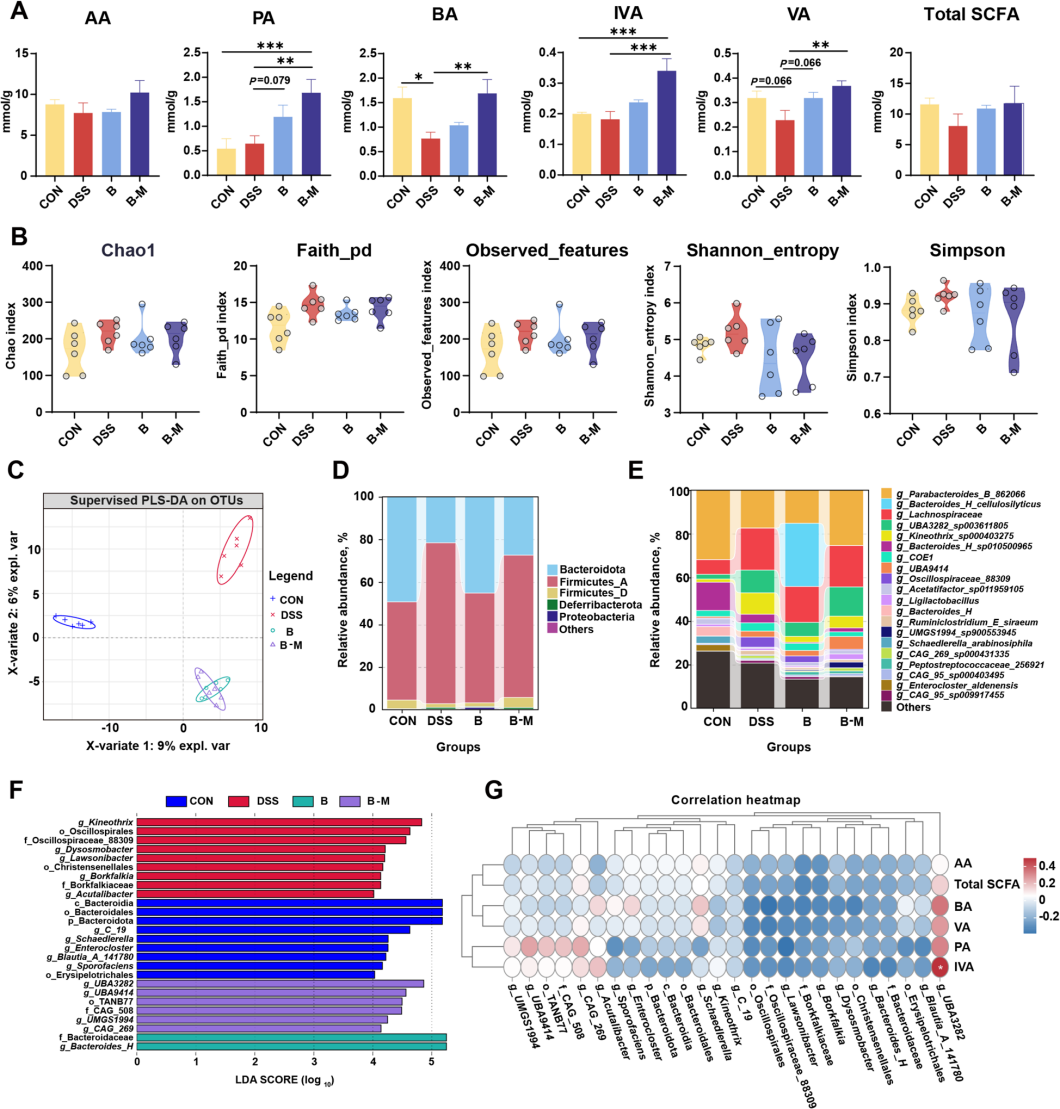
Effects of *B. hominis* LYH1 and metabolites on SCFA levels and gut microbiota in DSS-Colitis mice. **A** SCFA concentrations in colonic contents (*n* = 6). *AA* Acetic acid, *PA* Propionic acid, *BA* Butyric acid, *IVA* Isovaleric acid, *VA *Valeric acid, *Total SCFA *Total short-chain fatty acids. **B** α-Diversity indices (Chao1, Faith_PD, Observed_features, Shannon_entropy, Simpson). *n* = 6. **C** PLS-DA scores plot illustrating β-diversity of gut microbiota at the genus level (*n* = 6). **D** Relative abundance of gut microbiota at the phylum level for each treatment group. **E** Relative abundance of the top 20 gut microbial taxa at the genus level. **F** LEfSe analysis of gut microbiota communities at the genus level, highlighting significant taxa with LDA > 4. **G** Heatmap representing Spearman correlations between differential genera and SCFA concentrations. CON Control, DSS Administered 3% DSS, B Treated with 3% DSS and *B. hominis *LYH1 at a concentration of 10^9^ CFU/mL, B-M Exposed to 3% DSS alongside metabolites derived from *B. hominis *LYH1 at an equivalent concentration. Significant correlations are indicated by asterisks: *P < 0.05, **P < 0.01, and ***P < 0.001

By quantifying the concentrations of SCFAs in the colonic digesta, we observed significant elevations in the concentrations of propionic acid, butyric acid, valeric acid, and isovaleric acid in the colonic digesta of B-M mice compared to DSS mice (Fig. 6A, *P* < 0.05). Notably, PA and IVA levels were also greater in the B-M group compared to the CON group (Fig. 6A, *P* < 0.05). As SCFAs are critical metabolites produced by gut microbiota, we further investigated the microbial composition in the colonic digesta in these animals using 16S rRNA amplicon sequencing. Diversity indices, including Chao1, Faith_PD, Observed_features, Shannon, and Simpson, showed no significant differences between groups (Fig. 6B). Partial Least Squares Discriminant Analysis (PLS-DA) plots revealed a clear separation in microbial composition between the DSS and CON groups. Administration of live *B. hominis* LYH1 and its metabolites further induced notable alterations in the microbial composition (Fig. 6C). At the phylum level, the dominant taxa included Bacteroidota and Firmicutes, but no differences in the relative abundance of each phylum were found (Fig. 6D, *P* > 0.05). At the genus level, treated with live *B. hominis* LYH1 increased the relative abundance of *g_Bacteroides_H* compared to the DSS group (Fig. 6E, *P* < 0.05). Using LEfSe analysis (LDA > 4), we identified 26 differential microbial species across groups, with the highest number detected between the CON and DSS groups (Fig. 6F). Mice in group B exhibited five differential taxa, *g_UBA3282*, *g_UBA9414*, o_TANB77, f_CAG_508, *g_UMGS1994*, and *g_CAG_269*, and only two taxa, f_Bacteroidaceae and *g_Bacteroides_H*, were enriched in the colon of mice in group B-M. Spearman correlation analysis further demonstrated a significant positive correlation between the relative abundance of *g_UBA3282* in group B-M and IVA concentration (Fig. 6G).

## Discussion

Belonging to the Trichosporonaceae family, the genus *Blautia* has attracted special attention since its discovery due to its role in alleviating inflammatory and metabolic diseases, its antimicrobial activity against specific microorganisms, and its engagement in biotransformation and interaction with other gut microbes [28–31]. Despite demonstrating a range of potential probiotic attributes, a comprehensive understanding of *Blautia* remains elusive [32, 33], thereby necessitating thorough biological analysis for optimal utilization.

In this study, we successfully isolated a *B. hominis*-like strain from the intestinal tract of healthy pigs and established its novelty as *B. hominis* LYH1 through 16S rRNA analysis. Phenotypic identification indicated that the strain is devoid of hemolysin production and motility, traits that tentatively suggest a possible lack of virulence. Hemolysin, a pivotal virulence factor in bacterial pathogenesis, is capable of lysing host blood cells [34]. Bacterial motility, facilitated by flagella, is instrumental in processes such as migration toward intestinal villi, adhesion, biofilm formation, and virulence factor secretion [35]. The absence of both hemolytic activity and motility in *B. hominis* LYH1 suggests that it may pose minimal safety risks to hosts.

SCFAs play a crucial role in influencing the host's intestinal ecology, health, and nutrient metabolism [36]. In our experiment, a significant increase in the content of acetic acid and isovaleric acid was observed in *B. hominis* LYH1 after 72 h of fermentation in BHI medium, coupled with an almost threefold surge in total SCFA production, which provides strong evidence that *B. hominis* LYH1 is endowed with the ability to generate SCFAs. Even though a homogeneous medium was employed, genomic analysis unveiled that *B. hominis* LYH1 harbors five categories of CAZyme genes, among which GH families are the most prevalent, especially GH2 and GH109. The GH2 family comprises enzymes like β-galactosidase, β-mannosidase, β-glucuronidase, α-L-arabinosidase, mannoprotein-β-mannosidase, and others, capable of acting on diverse glycosidic bonds, encompassing β-1,4-glucosidic, β-1,3-glucosidic, and β-1,3-galactosidic bonds. Members of the GH109 family are typically linked to the degradation of plant polysaccharides, particularly intricate polysaccharides present in plant cell walls, such as cellulose, hemicellulose, and pectin. Metabolome analysis of *B. hominis* LYH1 confirmed that its genome contains various CAZymes, including α-glucosidase, α-galactosidase, and α-mannosidase, suggesting its capacity to degrade plant polysaccharides. These findings imply that *B. hominis* LYH1 possesses robust capabilities for metabolizing polysaccharides. These results collectively suggest that *B. hominis* LYH1 may utilize complex carbohydrates to produce SCFAs beneficial to the host.

Genome-wide analysis has elucidated that *B. hominis* LYH1 harbors an extensive repertoire of over ten drug resistance genes. Antibiotic susceptibility testing results demonstrate that this strain displays sensitivity to specific antibiotics, such as enrofloxacin, sulfadiazine, rifampicin, polymyxin, and tetracycline. Despite ongoing discourse surrounding antibiotic resistance, the prevalence of resistance genes in probiotics is not uncommon. For instance, most *Lactobacillus* species exhibit multidrug resistance to antibiotics like vancomycin, ciprofloxacin, cefotaxime, ceftriaxone, cefazolin, and aminoglycosides [37]. These resistance genes can be viewed as an outcome of bacterial-environment interactions, emerging from prolonged evolutionary processes and serving as a survival mechanism for bacteria. Of greater concern than the mere presence of resistance genes is their potential transferability [38]. Beyond resistance genes, *B. hominis* LYH1 produces a variety of antibiotic analogs, including Methyl 3,4-*O*-isopropylidene-D-lysine (also known as Methyl 3,4-*O*-isopropylidene-L-threonate) and 4-acetoxyphenol. *Blautia* species typically possess the ability to produce bacteriocins, which are evolutionary adaptations enabling bacteria to combat other microorganisms within their environment. In a previous study, *Blautia producta* inhibited vancomycin-resistant enterococci (VRE) growth by secreting antibiotic-like bacteriostats [39]. Consequently, the bacteriocin-producing ability of *B. hominis* LYH1 implies its potential to hinder gut colonization by pathogenic bacteria and modulate gut microbiota composition, possibly acting as a probiotic. This characteristic may partially explain the experimentally observed inhibitory effects on *E. coli* growth in co-culture studies.

Macrophages play a crucial role as the primary safeguard for intestinal immune homeostasis. They can be activated by diverse signals and differentiate into two distinct subtypes, classically activated (M1) and alternatively activated (M2) [40, 41]. M1 macrophages are stimulated by pro-inflammatory factors such as LPS or IFN-γ. Once activated, they secrete IL-6 and IL-12 to combat pathogens. However, the over-activation may lead to exacerbation of tissue damage [42]. Conversely, M2 macrophages are triggered by anti-inflammatory factors like IL-4 and IL-13. These macrophages secrete IL-10 and TGF-β to mediate anti-inflammatory responses and contribute to tissue repair [43–47]. In patients with IBD, intestinal damage and bacterial invasion are frequently accompanied by the polarization of macrophages towards the M1-activated inflammatory phenotype [48]. This characteristic makes macrophages a potential therapeutic target for the treatment of IBD [49]. *S.* Typhimurium has the ability to invade mononuclear phagocytes within host liver, spleen, lymph nodes, and Peyer's patches, where it replicates intracellularly. This bacterium is responsible for causing infectious gastroenteritis and systemic infections [50, 51]. Macrophages recognize Gram-negative bacteria, such as *S.* Typhimurium, through the binding of LPS to TLR4 [52]. During M1 polarization, macrophages induce the expression of inducible NOS2, which produces NO to eliminate pathogens [53, 54]. However, *S.* Typhimurium has evolved immune evasion strategies. For instance, it activates the STAT3 signaling pathway via the SarA (*Salmonella* anti-inflammatory response activator) effector. This activation promotes both intracellular replication and the production of IL-10 [54, 55]. To study *B. hominis* LYH1’s immunomodulatory effects on mouse macrophages, we created an in vitro inflammatory infection model using fluorescently labeled *S.* Typhimurium-infected RAW264.7 cells. This model induced a strong inflammatory response, shown by increased mRNA levels of *Tnf*, *Il6*, and *Il17a* cytokines. *S.* Typhimurium also upregulated the expression of *Il10* and *Tgfb1* genes, possibly as an immune evasion tactic. Commensal bacteria-exposed macrophages can activate immune responses [56], boosting immunity against common pathogens. Studies have demonstrated that *Lactobacillus plantarum* JL01 [57] and RS-09 [58] regulate M1 macrophage polarization and innate immune response to *S.* Typhimurium infection. Autophagy, through xerophagy-specific lysosomes, selectively captures and degrades intracellular bacteria, crucial for pathogen clearance [59]. Cytokines like IL-17A and IL-17F regulate autophagy, enhancing *Mycobacterium tuberculosis* elimination and autophagic activity in RAW264.7 cells [60]. Our study found that *B. hominis* LYH1’s live bacteria and metabolites inhibited *S.* Typhimurium replication and reduced inflammatory cytokine levels (*Tnf*, *Il17a*, *Il6*, and *Nos2*), likely due to reduced M1 polarization and attenuated inflammation. However, the exact mechanisms need further exploration.

In our cellular assays, metabolites from *B. hominis* LYH1 showed anti-inflammatory effects and enhanced cellular antimicrobial abilities without cytotoxicity. However, the gastrointestinal environment can affect probiotic activity in the body, so animal models are crucial for testing these bacteria's potential to reduce inflammation. IBD represents a prevalent intestinal disorder that significantly compromises the quality of life and growth in both human and animals. Existing treatment options are rather restricted, frequently depending on powerful yet potentially toxic medications. Prolonged use of such drugs further heightens the risks of infection [56, 61]. Despite the exact etiology of IBD remaining elusive, the dysregulation of the gut microbiota-immune system axis is regarded as a pivotal factor [62]. Recent investigations have pinpointed potential probiotics, among which are those sourced from the National Genomics Prediction database. These probiotics may enhance intestinal barrier function through modulating the microbiota, regulating immune responses, and activating regulatory T cells [63]. Our study aimed to create a colitis model in mice using DSS and test *B. hominis* LYH1’s effects on intestinal inflammatory injury, and the results were interesting. We found that while *B. hominis* LYH1 did not significantly affect weight loss or DAI scores, it reduced colon shortening caused by DSS, showing therapeutic potential.

In intestinal cells, barrier proteins like TJP1, OCLN, and CLDN1 are crucial for maintaining the gut barrier, preventing harmful substances from crossing [64]. Disrupting this barrier leads to immune issues and inflammation [65] DSS damages intestinal tight junctions, causing autoimmunity and microbial invasion [58]. Our study found that the gavage administration of live *B. hominis* LYH1 or its metabolites increased the abundance of *Tjp1*, exhibiting a repairing effect on the intestinal physical barrier.

IBD is marked by immune cells and lymphocytes buildup in the gut [66]. Our findings indicated that DSS-induced colitis caused a lasting immune response in mice, with greater blood lymphocyte counts. IBD patients have extensive macrophage infiltration in the gut, mainly from monocytes recruited by chemokines like *Ccl7* and *Ccl8* produced by intestinal macrophages [67]. Interestingly, DSS-induced colitis reduced blood monocyte and eosinophil levels, likely due to migration to inflamed tissues. Reduced red blood cell counts in inflamed mice may be attributed to acute anemia caused by intestinal hemorrhage. However, giving live *B. hominis* LYH1 increased eosinophil levels in colitis mice. Blood biochemical changes often reflect inflammation. For example, activated C3 drives inflammation [68], high TP levels indicate hemoconcentration from decreased serum water content (e.g., severe dehydration from diarrhea), low ALB and high bile acids suggest liver dysfunction [69]. Changes in immunoglobulin concentrations help diagnose infections, with high IgM levels indicating a recent infection [70]. In our study, DSS treatment increased serum C3, IgM, and TP levels, while decreasing ALB and triglyceride levels. Flow cytometry showed that DSS-induced colitis increased T lymphocytes (CD3^+^), helper T cell (CD3^+^CD4^+^), and cytotoxic T cell (CD3^+^CD8^+^) proportions in the blood, indicating systemic T cell activation. CD8^+^ T cells release serine proteases and pore-forming cytolytic proteins to kill target cells, while CD4^+^ T cells orchestrate the immune response by secreting cytokines [68]. Imbalance in T-lymphocyte subpopulations in IBD patients causes ongoing tissue damage. Recent studies link cytotoxic CD8^+^ T cells (Tc1) and IL-17-producing CD8^+^ T cells (Tc17) to IBD, with dual expression of IFN-γ^+^ IL-17^+^ and Foxp3^+^ IL-17^+^ in CD8^+^ T cells [71]. This altered ratio of CD8^+^ T cells highlights T cell plasticity [72]. Our results suggest that gavage administration of *B. hominis* LYH1 can mitigate intestinal inflammatory responses by reducing T-lymphocyte subpopulation activation in peripheral blood.

To further explore how *B. hominis* LYH1 protects against colonic barrier damage in mice, we examined the gene expression levels of cytokines and signaling molecules in the intestines. M1 macrophages, marked by NOS2, CXCL10, and NLRP3, are known for high pro-inflammatory cytokine expression like TNF-α, IL-1β, and IL-6 [73]. In contrast, M2 macrophages, marked by anti-inflammatory markers like IL-10, Arg1, Retnla, Chil3, and CD163 [73], help resolve inflammation. Chemokines such as CCL17, CCL22, and CCL2 play key roles in recruiting monocytes and macrophages to inflammatory sites [74, 75]. Gene expression analysis showed that pro-inflammatory genes linked to M1 macrophages were highly expressed in the DSS-induced colitis model, indicating strong inflammatory responses. However, *B. hominis* LYH1 live bacteria and metabolites significantly reduced the expression of pro-inflammatory factors, suggesting that *B. hominis* LYH1 alleviates intestinal inflammation in mice by affecting M1-type macrophage polarization.

We have confirmed that *B. hominis* LYH1 can produce SCFAs in BHI medium. SCFAs positively affect host immune cell differentiation and metabolic processes, thereby regulating susceptibility to pathogenic bacteria [76]. The most common SCFAs in the gut of human and animals, acetic acid, propionic acid, and butyric acid, are involved in immunomodulation and anti-inflammatory responses [77]. This study found that oral administration of *B. hominis* LYH1 metabolites enhanced intestinal immunomodulation and anti-inflammatory capacity in mice, which may be mediated by changes in gut microbial composition and increased SCFA levels. Notably, *B. hominis* LYH1 did not alter intestinal microbial diversity in mice despite increased SCFA production. This paradoxical finding suggests that probiotics may have a greater impact on gut function rather than altering the compositional structure of the gut microbiome [78]. This is supported by a previous study showing that human ingestion of *Lactobacillus suis* Bar13 and *Bifidobacterium longum* Bar33 significantly increased acetic acid and valeric acid levels without altering fecal microbiota structure [79]. Consequently, further research is essential to understand the effects of *B. hominis* LYH1 on gut microbial community and its function.

## Conclusion

The biological properties of *B. hominis* LYH1 render it a highly promising candidate for probiotic applications and therapeutic interventions in maintaining gut health. This strain exhibits remarkable antimicrobial activity against *E. coli* ATCC 25922. Genomic analysis uncovers the existence of critical CAZyme genes, such as those encoding α-glucosidase, α-galactosidase, and α-mannosidase, highlighting its significance in intestinal carbohydrate metabolism. Functionally, both the cells of *B. hominis* LYH1 and its metabolites effectively inhibit the intracellular replication of *S.* Typhimurium in macrophages. They reduce the infection rate and mitigate cellular inflammatory damage by modulating macrophage polarization and downregulating the expression of pro-inflammatory cytokine genes. These anti-inflammatory effects are further validated in a murine colitis model, wherein oral administration of *B. hominis* LYH1 or its metabolites suppresses the expression of pro-inflammatory cytokines, attenuates T-cell activation, and alleviates systemic inflammation. Furthermore, *B. hominis* LYH1 plays a role in maintaining gut homeostasis by promoting the production of SCFAs, which are essential regulators of intestinal health. These findings indicate that *B. hominis* LYH1 could be a potential candidate for further research on probiotics and the development of therapeutic strategies aimed at gut inflammation and modulating the microbiota.

## Supplementary Information

Additional file 1: Fig. S1 Whole-Genome Analysis of *B. hominis* LYH1. A GO (Gene Ontology, http://www.geneontology.org) annotation of *B. hominis* LYH1. B COG (Clusters of Orthologous Groups of proteins) functional gene classification of *B. hominis* LYH1. C Species composition of *B. hominis* LYH1 carbohydrate enzyme gene. Table S1 Evaluation of disease activity index. Table S2 Primer sequences used in the experiment. Table S3 Phenotypic characteristics of *B. hominis* LYH1. Table S4 Drug resistance gene types of *B. hominis* LYH1. Table S5 Antibiotic analogs in exclusive metabolites of *B. hominis* LYH1. Table S6 Inhibitory zone diameters of antibiotics against *B. hominis* LYH1.

## Data Availability

The datasets used during the current study are available from the corresponding author on reasonable request.

## Abbreviations

ALB: Albumin
AA: Acetic acid
AAs: Auxiliary activities
Arg1: Arginase 1
AB: Alcian blue
AAMs(M2): Alternatively activated macrophages (M2 subtype)
BHI: Brain heart infusion
BA: Butyric acid
CD: Crohn's disease
CRC: Colorectal cancer
CTAB: Cetyltrimethylammonium bromide
CAZymes: Carbohydrate active enzymes
C3: Complement C3
CRP: C-reactive protein
COG: Clusters of orthologous groups of proteins
CEs: Carbohydrate esterases
Cd86: Cluster of differentiation 86
Cxcl10: C-X-C motif chemokine ligand 10
Ccl2: C-C motif chemokine ligand 2
Chil3: Chitinase-like 3
Cd163: Cluster of differentiation 163
Ccl17: C-C motif chemokine ligand 17
Ccl22: C-C motif chemokine ligand 22
Cldn1: Claudin 1
CAMs(M1): Classically activated macrophages (M1 subtype)
DSS: Dextran sodium sulfate
DAI: Disease activity index
Eos: Eosinophil
FGPs: First-generation probiotics
GAM: Gifu anaerobic medium
Gapdh: Glyceraldehyde-3-phosphate dehydrogenase
GHs: Glycoside hydrolases
GTs: Glycosyltransferases
GO: Gene Ontology
HGB: Hemoglobin
HCT: Hematocrit
HE: Hematoxylin and eosin
IBD: Inflammatory bowel diseases
IgG/M: Immunoglobulin-G/M
IsoVA: Isovaleric acid
Il6: Interleukin-6
Il1b: Interleukin-1beta
Il10: Interleukin-10
Il17a: Interleukin-17A
Ifng: Interferon gamma
KEGG: Kyoto Encyclopedia of Genes and Genomes
LB: Luria-bertani
Lym: Lymphocyte
LDH: Lactate dehydrogenase
LEfSe: Linear discriminant analysis of effect size
LPS: Lipopolysaccharide
Mon: Monocyte
MFI: Mean fluorescence intensity
NGPs: Next-generation probiotics
NCBI: National Center for Biotechnology Information
Neu: Neutrophil
NR: Non-Redundant Protein Database
Nos2: Nitric oxide synthase 2
Nlrp3: NLR family, pyrin domain containing 3
Ocln: Occludin
PLT: Platelet
PA: Propionic acid
Pfam: Protein family database
PLs: Polysaccharide lyases
PCA: Principal component analysis
PLS-DA: Partial least squares discriminant analysis
PBS: Phosphate-buffered saline
RFP: Red fluorescent protein
RBC: Red blood cell
Retnla: Resistin-like alpha
SCFAs: Short-chain fatty acids
TP: Total protein
TG: Triglycerides
TCH: Total cholesterol
TBA: Total bile acids
Tnf: Tumor necrosis factor-alpha
Tgfb1: Transforming growth factor-beta 1
Tjp1: Tight junction protein 1
Tregs: Regulatory T cells
VA: Valeric acid
VRE: Vancomycin-resistant enterococci
WBC: White blood cell
