# Supplementary material for: Unlocking the power of swine gut bacteria: newly isolated Blautia strain and its metabolites inhibit the replication of Salmonella Typhimurium in macrophages and alleviate DSS-induced colitis in mice
Source: J Anim Sci Biotechnol. 2025 Jun 23;16:87. doi: 10.1186/s40104-025-01208-7 (PMC12183824; doi:10.1186/s40104-025-01208-7)
Supplement: Supplementary file 1 — Additional file 1: Fig. S1 Whole-Genome Analysis of B. hominis LYH1. A GO (Gene Ontology, http://www.geneontology.org) annotation of B. hominis LYH1. B COG (Clusters of Orthologous Groups of proteins) functional gene classification of B. hominis LYH1. C Species composition of B. hominis LYH1 carbohydrate enzyme gene. Table S1 Evaluation of disease activity index. Table S2 Primer sequences used in the experiment. Table S3 Phenotypic characteristics of B. hominis LYH1. Table S4 Drug resistance gene types of B. hominis LYH1. Table S5 Antibiotic analogs in exclusive metabolites of B. hominis LYH1. Table S6 Inhibitory zone diameters of antibiotics against B. hominis LYH1. [file 40104_2025_1208_MOESM1_ESM.docx]

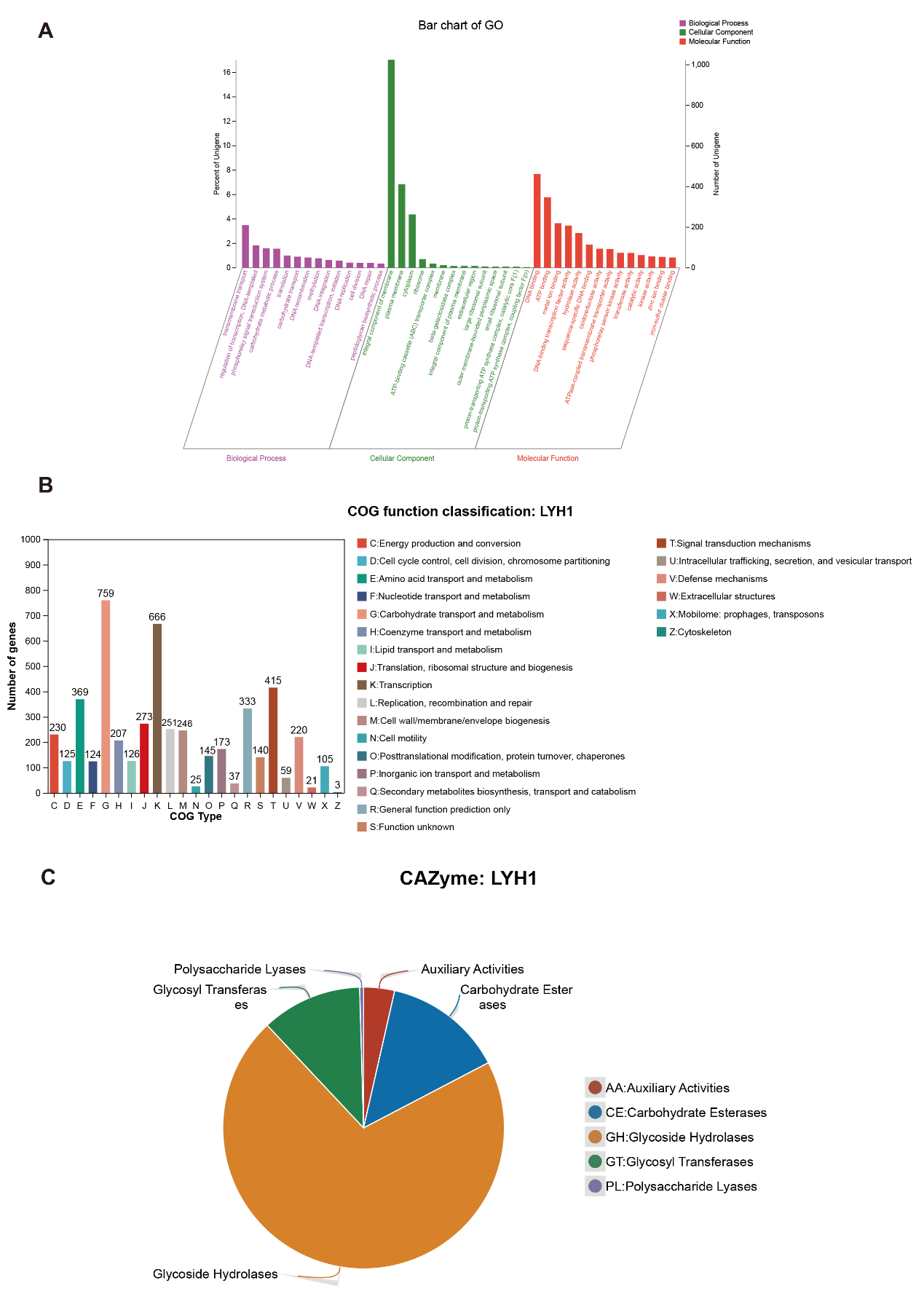
**Supplementary materials**

**Fig. S1** Whole-Genome Analysis of *B. hominis* LYH1. **A** GO (Gene Ontology, http://www.geneontology.org) annotation of *B. hominis* LYH1. **B** COG (Clusters of Orthologous Groups of proteins) functional gene classification of *B. hominis* LYH1. **C** Species composition of *B. hominis* LYH1 carbohydrate enzyme gene

**Table S1** Evaluation of disease activity index (DAI^1^)

| **Decrease of body weight, %** | **Stool characteristics** | **Stool occult blood** | **Score** |
| --- | --- | --- | --- |
| 0 | Normal Stools | Normal Stools | 0 |
| 1-5 | Loose stools | Positive for occult blood | 1 |
| 5-10 | Loose stools | Positive for occult blood | 2 |
| 10-15 | Watery stools | Stools with visible blood | 3 |
| >15 | Watery stools | Stools with visible blood | 4 |

^1^DAI= (weight loss rate + fecal characteristics + bleeding situation)/3

**Table S2** Primer sequences used in the experiment

| **Targeting gene** | **Primer sequence （5’ to 3’）** |
| --- | --- |
| *Gapdh* | F: CTCCCACTCTTCCACCTTCG |
|  | R: CCACCACCCTGTTGCTGTAG |
| *Actb* | F: GTCGGAGTGAACGGATTTGG |
|  | R: CAATGTCCACTTTGCCAGAGTTAA |
| *Il6* | F: TACCACTTCACAAGTCGGAGGC |
|  | R: CTGCAAGTGCATCATCGTTGTTC |
| *Il10* | F: CGGGAAGACAATAACTGCACCC |
|  | R: CGGTTAGCAGTATGTTGTCCAGC |
| *Tnf* | F: GGTGCCTATGTCTCAGCCTCTT |
|  | R: GCCATAGAACTGATGAGAGGGAG |
| *Il17a* | F: TCAGCGTGTCCAAACACTGAG |
|  | R: CGCCAAGGGAGTTAAAGACTT |
| *Nos2* | F: CAGGTCTTTGACGCTCGGAA |
|  | R: GCCTGAAGTCATGTTTGCCG |
| *Tgfb1* | F: CCACCTGCAAGACCATCGAC |
|  | R: CTGGCGAGCCTTAGTTTGGAC |
| *Ocln* | F: GCAATGACATGTATGGCGGAG |
|  | R: TGTCCCAAGCAAGTGTGGAA |
| *Cldn1* | F: TTTGGCCAGGCCCTCTTTAC |
|  | R: AGGTTGTTTTCCGGGGACAG |
| *Tjp1* | F: GAGCCCCCTAGTGATGTGTG |
|  | R: CCAACCGTCAGGAGTCATGG |
| *Il1b* | F:AACCTGCTGGTGTGTGACGTTC |
|  | R:CAGCACGAGGCTTTTTTGTTGT |
| *Ifng* | F: AGCAAGGCGAAAAAGGATGC |
|  | R: TCATTGAATGCTTGGCGCTG |
| *Cd86* | F: ACGTATTGGAAGGAGATTACAGCT |
|  | R: TCTGTCAGCGTTACTATCCCGC |
| *Cd163* | F: ATCCTCGGGGGTCATTCAGA |
|  | R: GCCTGGGCTCTTGTTCCATT |
| *Cxcl10* | F: CCAAGTGCTGCCGTCATTTTC |
|  | R: GGCTCGCAGGGATGATTTCAA |
| *Nlrp3* | F: ATTACCCGCCCGAGAAAGG |
|  | R: TCGCAGCAAAGATCCACACAG |
| *Arg1* | F: AGCACTGAGGAAAGCTGGTC |
|  | R: CAGACCGTGGGTTCTTCACA |
| *Retnla* | F: TCCAGCTGATGGTCCCAGTGAATA |
|  | R: ACAAGCACACCCAGTAGCAGTCAT |
| *Ccl17* | F: CAAGCTCATCTGTGCAGACC |
|  | R: CGCCTGTAGTGCATAAGAGTCC |
| *Ccl22* | F: AAGACAGTATCTGCTGCCAGG |
|  | R: GATCGGCACAGATATCTCGG |
| *Ccl2* | F: CCACTCACCTGCTGCTACTCA |
|  | R: TGGTGATCCTCTTGTAGCTCTCC |
| *Chil3* | F: TACTCACTTCCACAGGAGCAGG |
|  | R: CTCCAGTGTAGCCATCCTTAGG |

**Table S3** Phenotypic characteristics of *B. hominis* LYH1

| **Item** | ***Blautia hominis LYH1*** |
| --- | --- |
| Gram staining | + |
| Spore staining | - |
| Moveability | - |
| Catalase activity | - |
| Activity of oxidase | - |
| Tactile enzyme activity | - |
| Hemolytic activity | - |

**Table S4** Drug resistance gene types of *B. hominis* LYH1

| **Drug types** | **Numbers** |
| --- | --- |
| Macrolide antibiotic | 116 |
| Tetracycline antibiotic | 86 |
| Fluoroquinolone antibiotic | 70 |
| Glycopeptide antibiotic | 69 |
| Penam | 63 |
| Aminoglycoside antibiotic | 31 |
| Phenicol antibiotic | 26 |
| Acridine dye | 25 |
| Aminocoumarin antibiotic | 23 |
| Cephalosporin | 23 |
| Lincosamide antibiotic | 23 |
| Cephamycin | 23 |
| Streptogramin antibiotic | 19 |
| Pleuromutilin antibiotic | 16 |
| Carbapenem | 15 |
| Monobactam | 14 |
| Oxazolidinone antibiotic | 14 |
| Glycylcycline | 12 |
| Nitroimidazole antibiotic | 12 |

**Table S5** Antibiotic analogs in exclusive metabolites of *B. hominis* LYH1

| **Name** | **Structural formula** | **Fold change** | **Super class** |
| --- | --- | --- | --- |
| Methyl 3,4-o-isopropylidene-L-threonate | C_8_H_14_O_5_ | 4.82 | Organic oxygen compounds |
| 4-Acetoxyphenol | C_8_H_8_O_3_ | 2.10 | Undefined |
| 5-Methylbenzotriazole | C_7_H_7_N_3_ | 1.67 | Undefined |
| Pyripyropene a | C_31_H_37_NO_10_ | 1.31 | Lipids and lipid-like molecules |
| Gatifloxacin | C_19_H_22_FN_3_O_4_ | 1.28 | Organoheterocyclic compounds |

**Table S6** Inhibitory zone diameters of antibiotics against *B. hominis* LYH1

| **Antibiotics** | **Titer, μg** | **Inhibitory Zone Diameter (IZD), mm** | **Sensitivity** |
| --- | --- | --- | --- |
| Amoxicillin | 10 | 0 | - |
| Enrofloxacin | 5 | 20 | + |
| Sulfadiazine | 300 | 30 | + |
| Streptomycin | 10 | 0 | - |
| Rifampicin | 5 | 55 | + |
| Polymyxin | 300 | 23 | + |
| Tetracycline | 30 | 24 | + |
| Clindamycin | 2 | 0 | - |
| Ampicillin | 20 | 32 | + |
| Linezolid | 30 | 56 | + |
| Cefepime | 30 | 0 | - |
| Imipenem | 10 | 0 | - |
| Clarithromycin | 15 | 0 | - |
| Vancomycin | 30 | 18 | + |
| Gentamicin | 10 | 0 | - |
